# The Mental Health of Sporting Officials: A Systematic Review and Meta-analysis

**DOI:** 10.1007/s40279-025-02315-1

**Published:** 2025-09-27

**Authors:** Riki Lindsay, Courtney C. Walton, Aden Kittel, Dominic G. McNeil, Paul Larkin, Michael Spittle, Suzanne M. Cosh

**Affiliations:** 1 Collaborative Evaluation and Research Centre, Federation University, Ballarat, Australia; 2 Melbourne School of Psychological Sciences, University of Melbourne, Melbourne, Australia; 3 Centre for Sport Research, Institute for Physical Activity and Nutrition, School of Exercise and Nutrition Sciences, Deakin University, Geelong, Australia; 4 Institute for Health and Sport, Victoria University, Melbourne, Australia; 5 MSA Research Centre, Maribyrnong Sports Academy, Melbourne, Australia; 6 School of Psychology, University of New England, Armidale, Australia; 7 Australian Football League, Melbourne, Australia; 8 School of Psychology, University of Adelaide, Adelaide, Australia

## Abstract

**Background:**

The mental health of participants in sport has attracted increasing focus within research, practice, and policy. While mental health in sports has received increased attention, the mental health of sporting officials—such as judges, referees, and umpires—remains significantly under-researched compared with athletes. To our knowledge, there are no systematic reviews and meta-analyses that have synthesised available prevalence data of mental health symptoms and disorders in sporting officials. In addition, while previous reviews have provided a broad overview of risk and protective factors in relation to overall mental health, links between identified factors and specific mental health and well-being outcomes have not been explored. Therefore, this study aimed to systematically review and analyse the prevalence rates of mental health symptoms and disorders (e.g., depression and anxiety) among sporting officials and identify specific risk and protective factors influencing sporting officials’ mental health and/or psychological well-being.

**Methods:**

Relevant studies were retrieved from SCOPUS, Web of Science, SPORTDiscus and PsycINFO up until July 2025. Prevalence rates of specific mental health outcomes (i.e. anxiety and depressive symptoms) were meta-analysed.

**Results:**

A total of 26 studies were included. Meta-analyses comprising 2797 sporting officials showed that the pooled proportion of elevated anxiety symptoms was 19.1% (95% CI 13.4–27, *I*^2^ = 94.1%) and 20.6% (95% CI 12.4–32.3, *I*^2^ = 97.3%) for elevated symptoms of depression. Sport-environmental risk factors were identified in 70% of the studies (*k* = 19) (e.g., levels of professional experience, environment around matches, experiences of abuse), while 48% of studies (*k* = 13) examined personal risk factors (e.g., age, sex, injury). A total of 37% of studies (*k* = 10) examined sport-environmental protective factors (e.g., years of officiating experience, level of officiating, hours and frequency of officiating), while 33% of studies (*k* = 9) investigated personal protective factors (e.g., emotional intelligence, feelings of competence, age, sex).

**Conclusions:**

The results suggest that targeting change at various levels of the sport ecosystem may help foster and promote positive mental health outcomes among sporting officials. The findings of this review suggest that strategies tailored to officials could include age/level of experience-specific support interventions and creating organisational cultures that prioritise mental health outcomes.

**Supplementary Information:**

The online version contains supplementary material available at 10.1007/s40279-025-02315-1.

## Key Points

**Table:** 

Sporting officials face significant mental health challenges, with our results indicating a pooled prevalence rate of 19% for elevated anxiety symptoms and 21% for elevated symptoms of depression.
Key risk factors for poor mental health include age (younger officials), lower years of officiating experience and experiences of verbal/physical abuse. Protective factors include more years of officiating experience, officiating at higher levels of competition and feelings of competence.
Findings from this review highlight the need for targeted interventions and organisational changes to facilitate positive mental health outcomes among sporting officials.

## Introduction

The mental health of participants in sport has attracted increasing focus within research, practice and policy. There is now significant evidence suggesting rates of poor mental health among athletes, coaches and high-performance staff that mirror if not slightly surpass community norms [1–4]. Within the sporting context, various stressors—such as performance pressure and organisational demands—can contribute to poor mental health (see Sarkar and Fletcher [5] for a comprehensive review). Officials (e.g. judges, referees and umpires) represent a vital stakeholder group within sport alongside athletes, coaches and high-performance staff. Though officials and athletes experience several similar stressors in sport (e.g. physical demands, competitive pressures), research suggests that officials may experience a range of additional stressors that may contribute to poor mental health outcomes including verbal and physical abuse, making the wrong decision, being out of position and/or being in the way and environmental pressure (e.g. spectators) to let matches flow [6, 7]. Despite an increased focus on mental health in sports, sporting officials’ mental health remains under-researched in comparison to athletes.

Sporting officials' attrition rates are high worldwide, with reports indicating that between 20 to 35% of sports officials of various levels of competition and sports do not return after a single season [8]. In addition to competition stressors, a range of non-competition-based factors can lead to officials’ attrition. For example, quality of their social support and communities [9], and negative social experiences [10, 11] are among the reported reasons that can contribute to attrition rates. Negative experiences in these areas may also contribute to reduced well-being and mental health outcomes [10, 11]. Further, it has been reported that the high-pressure environments in which sporting officials operate, combined with the other environmental factors (e.g. lack of perceived organisational support), puts them at significant risk of poor mental health, such as elevated symptoms of anxiety, depression and burnout [7].

A critical component of promoting the mental health of sporting officials is to identify key personal and environmental factors experienced by officials, and more clearly identifying how they influence mental health (e.g. anxiety and depressive symptoms) and well-being (e.g. subjective/psychological well-being domains, life satisfaction) outcomes. In environments where officials’ personal needs and motives align with available support, protective factors such as emotional intelligence, social support and organisational backing can strengthen the connection between an official and their environment, fostering resilience and mental well-being [7]. Conversely, when officials face excessive demands such as abuse, decision-making pressure and inadequate support, this misalignment contributes to poor mental health, including elevated anxiety and depressive symptoms and burnout. The distinction between these risk and protective factors allows practitioners and organisations to develop targeted prevention and intervention strategies. In turn, this may lead to a decrease in the attrition of officials. Though many sporting organisations prioritise mental health for athletes, there has been less focus in the literature to date regarding mental health support for officials [7]. One potential reason may be limited evidence around risk and protective factors contributing to specific mental health and well-being outcomes among sporting officials. In addition, officials have historically been regarded as an afterthought compared with athletes and coaches when decisions are made about funding, policy, professional development and research [12].

It is notable that two reviews have recently been published which examine mental health among sporting officials [7, 13]. In a recent scoping review, Carter et al. [7] identified poor mental health outcomes—such as anxiety, burnout and exposure to non-accidental violence—are influenced by factors such as gender, age and officiating experience. This review primarily provided an overview on mental health among officials and was an important step in developing an understanding of mental health in sport officials. However, this review was descriptive, and did not synthesise prevalence data, which are needed to better understand the current best estimates of prevalence in this population. Further, consistent with the purpose of a scoping review, a broad overview of risk and protective factors in relation to overall mental health was provided; however, associations between the identified factors and specific mental health outcomes were not explored. Synthesising these associations can help to understand the relationship of various risk/protective factors to specific mental health and well-being outcomes. The review of Mojtahedi et al. [13] was explicitly focused on officials’ experiences of abuse but did report abuse can have a negative impact on well-being for officials at all levels of competition. The current review builds on these reviews by combining a meta-analytical approach to prevalence data with a detailed examination of how varied factors are associated with specific mental health and well-being outcomes among officials. This review will help to inform future research, and support policy makers and sporting organisations in delivering effective mental health and well-being support systems for sporting officials. Notably, this review will build on a key recommendation from a recent expert statement on sport officiating research, by helping to identify the influences and determinants of mental health in this population [14].

This systematic review and meta-analysis aimed to build on the existing literature by systematically investigating both personal and environmental risk and protective factors related to specific mental health and well-being outcomes among sporting officials. Two specific research questions are to be addressed:

1. What are the prevalence rates of mental health symptoms and disorders (e.g. depression and anxiety) among sporting officials?
2. What are the risk and protective factors related to specific mental health (i.e. anxiety, depressive symptoms, positive affect) and well-being (i.e. subjective/psychological well-being domains, life satisfaction) outcomes among sporting officials?

In this review, mental health is conceptualised according to Keyes’ [15] two-continuum model, which distinguishes between two related, but distinct dimensions: mental health and mental illness. One continuum reflects the presence or absence of mental health, while the other the presence or absence of mental illness [15]. From this perspective, officials may simultaneously experience aspects of positive mental health alongside mental illness.

## Methods

The review was conducted in line with PRISMA guidelines [16] and in accordance with the process for systematic reviews in sport and exercise [17]. The review was registered with PROSPERO on 28 March 2024 (CRD42024523897). A small number of deviations from the original protocol were made, including the addition of meta-analyses of prevalence data and clarification of inclusion terms to reduce ambiguity of terminology (e.g., mental health vs mental health symptoms and disorders). These updates are recorded and registered with PROSPERO (CRD42024523897).

### Search Strategy

Electronic databases (SCOPUS, Web of Science, SPORTDiscus, PsycINFO) were initially searched in December 2023, repeated in December 2024, and again in July 2025 to identify additional published articles and ensure this review is as current as possible. Search terms included (‘mental’ AND ‘illness’ OR ‘mental’ AND ‘disorder’ OR ‘psych*’ AND ‘problem’ OR ‘depression’ OR ‘anxiety’ OR ‘stress’) OR (‘mental’ AND ‘health’ OR ‘mental’ AND ‘well-being’ OR ‘mental’ AND ‘well-being’ OR ‘flourishing’) AND (‘sport*’) AND (‘referee’ OR ‘umpire’ OR ‘official’). Boolean phrases were used for each string to ensure all potentially appropriate literature was captured during the search process. An initial draft of search terms was proposed by the lead author (R.L.) to the entire authorship team. Terms were then discussed as an entire team and further developed until consensus was reached on appropriate terms for inclusion.

### Inclusion and Exclusion of Studies

Eligible studies presented original quantitative data (including cross-sectional, longitudinal, retrospective audit or prospective designs) from any date range. Ineligible articles were those published in languages other than English, qualitative studies, narrative or systematic reviews or position statements.

I. Population*:* Studies were included if they reported on current sporting officials of any age and level of experience, defined as individuals engaged in officiating team or individual sports (i.e. referees, umpires and judges) [18]. Thus, activities such as dance or recreational fitness, and non-officiating roles, such as coaching or event management were excluded.
II. Interest*:* In line with previous reviews on mental health in officials and athletes [7, 19, 20], studies had to be related to mental health or ill-health (i.e. anxiety, depressive symptoms, positive affect) and well-being (i.e. subjective/psychological well-being domains, life satisfaction) outcomes among sport officials.
III. Context*:* Current sporting officials from any sport setting.
IV. Outcomes*:* Studies were eligible if they included valid assessment measures of mental health symptoms, mental health disorders (e.g. depression, anxiety) or psychological well-being (e.g. well-being, life satisfaction). Studies that focused exclusively on acute, transient psychological reactions or mood states (e.g. pre-competitive anxiety) that are not reflective of our broader focus on well-being or mental ill-health were excluded.

### Screening Strategy and Study Selection

Articles were screened independently by two authors (R.L. and A.K.). Both authors screened titles and abstracts using Covidence online systematic review software (Covidence, Veritas Health Innovation Ltd., Melbourne, Australia) and disagreements were resolved by a third author (D.M.). Following title and abstract review, both authors screened the full texts to assess eligibility for inclusion, with any discrepancies again resolved by a third author (D.M.).

### Data Extraction

One author (R.L.) completed data extraction using a customised data extraction form. One other author (D.M.) randomly audited the accuracy of extracted data from six of the included articles (20% of total articles included in review). Any discrepancies in the data extraction form were discussed with a third and fourth author (D.M., S.C.) until a consensus was reached. Data about the year of publication, country of participants, sport(s), sample size, sample characteristics such as officiating level and setting, study design, main outcomes and key findings were extracted. Where appropriate, data outlining mental health risks and/or protective correlates/associations were summarised and synthesised in the results.

### Procedure for Identifying Protective and Risk Factors for Officials’ Mental Health

To identify risk and protective factors, the current review adopted the procedure used in a recent review on mental health in elite athletes by Küttel and Larsen [20]. This involved classifying the correlates (e.g. age, sex, level of officiating experience) related to specific mental health (i.e. anxiety, depressive symptoms) and well-being (i.e. subjective/psychological well-being domains, life satisfaction, positive affect) outcomes for each of the included studies. Correlates were listed separately for studies that investigated multiple variables. Following this, the direction of relationships of the correlates was examined to categorise them as either protective or risk factors. Based on the previous literature (e.g. Küttel and Larsen [20]), correlates were grouped into overarching themes. For example, concerns about performance, thinking about past failures, and fear of failure were grouped into ‘negative content about performance.’ High emotional intelligence, transformational and developer leadership styles, higher levels of formal education, and higher levels of perceived role competence were grouped into ‘emotional and cognitive resources.’ Overarching protective and risk factor themes were divided into sport-environmental and personal domains.

### Critical Appraisal

In line with similar reviews on mental health in sport [21], study quality was assessed using the Joanna Briggs Institute (JBI) tools (https://jbi.global/critical-appraisal-tools). The JBI tools enable studies to be assessed against a list of criteria that is appropriate to the specific design of each study [22]. Two reviewers (M.S., P.L.) critically appraised each study, which was verified by one additional author (R.L.).

### Effect Measures and Data

Prevalence of mental health symptoms data was analysed using a random-effects meta-analysis of event rates, expressed as mental health symptoms prevalence rates (i.e. percentage of study sample)*,* conducted using Comprehensive Meta-analysis Version 4 [23]. The random-effects method was implemented because of its conservative summary estimate and the incorporation of between- and within-study variance [24]. This methodological approach has been implemented in similar research analysing mental health symptom prevalence in athlete populations [1]. Heterogeneity was evaluated using the *I*^*2*^ statistic and interpreted according to Cochrane guidelines [25]. To explore heterogeneity between studies further, a meta-regression was planned for continuous covariates when *k* ≥ 10 and sub-group analyses when *k* ≥ 2 per subgroup [21]. Potential reporting bias was assessed by inspection of the funnel plot and Egger’s trim-and-fill method [26].

## Results

### Study Selection

**Fig. 1:**
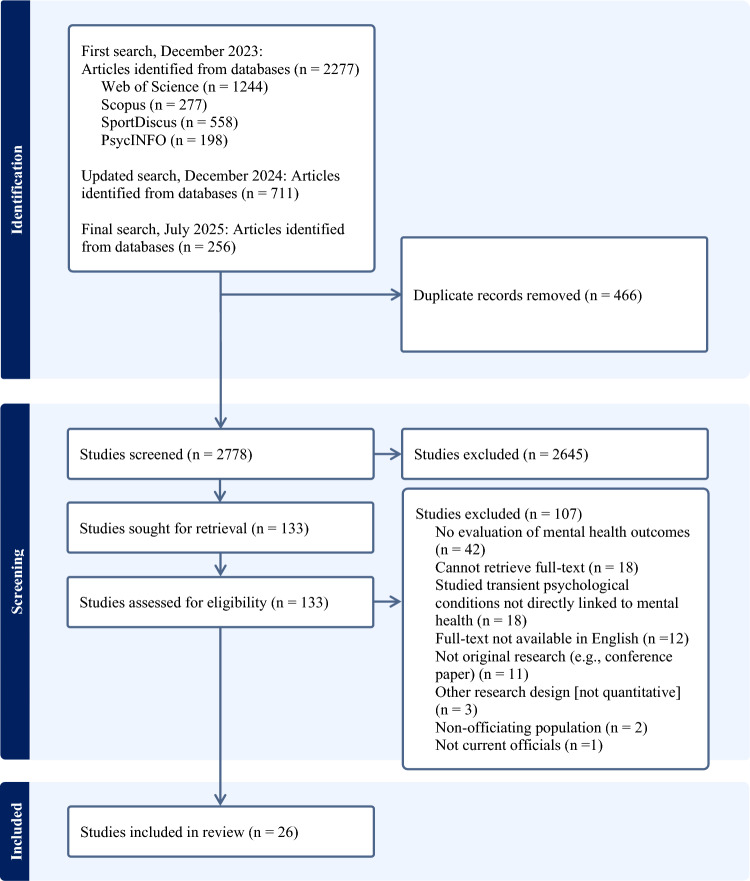
PRISMA study identification and selection flow chart

A total of 3244 articles were identified across four databases, with 2778 articles remaining after the removal of duplicates. After screening 133 full texts, 26 studies reporting on 25 datasets were included in the final review. Figure 1 displays the search and selection process.

### Study Characteristics

Key results are presented in Table 1. The total population for the current review was 11,618. In the studies identified, sample sizes ranged from 15 to 4099, with an average sample size of 447 officials. Of included studies, 76% of officials identified as male (*n* = 9084) and 10% as female (*n* = 1188). Three studies did not report gender. None of the included studies reported trans/intersex/other gender. Of the included studies, six focused on elite-level officials, with the remainder reporting on officials that were adjudicating at amateur (*k* = 4), elite/sub-elite (*k* = 4), sub-elite/amateur (*k* = 4), sub-elite (*k* = 3), elite/sub-elite/amateur (*k* = 2), elite/amateur (*k* = 2) and skill level not specified (*k* = 1). Included studies were predominately conducted in three regions—Europe (*k* = 7), Asia (*k* = 4) or North America (*k* = 4)—with further studies including officials from the UK (*k* = 4), South America (*k* = 1) and Australia (*k* = 1). The remaining studies comprised samples from multiple countries (*k* = 4). The most frequent sport investigated was soccer (*k* = 13), followed by basketball (*k* = 3), while two studies reported on officials in multiple sports. Six other sports were investigated in one study each (netball, volleyball, rugby union, rugby league, ice hockey, table tennis). One study did not specify a sport. Study quality varied (See Tables 2, 3).

**Table 1:** Characteristics of included studies

					Variables		Mental health/Wellbeing correlates/associations		
Study	Study design	Aims	*N* (female:male) Age of officials location/context	Sport(s) Level of officiating	Predictor(s)	Outcome(s)	Protective	Risk	Significant main findings/contributions
Al-Haramlah [27]	Cross-sectional descriptive analytical approach	Examine psychological burnout among international table tennis referees	20 (NR) Age range: 30–39 (*n* = 5, 25%), 40–49 (60%), 50 + (15%) Saudi-Arabia	Table Tennis International	Psychological pressure scale	Psychological burnout scale		Increased psychological pressure from in-match decision making	Positive correlation between psychological pressure and burnout frequency and intensity. Higher levels of psychological pressure stemming from decision-making may contribute to higher levels of burnout frequency and/or intensity
Alkhawaldeh and Madanat [28]	Cross-sectional	Examine levels of psychological stress in national soccer referees in the absence of spectators	300 (30:270) Age range: < 30 (16%), 30–39 (17%), 40–49 (34%), > 50 (30%) Jordan	Soccer National	Type of referee	COPSQQ		Role responsibilities-Responsibilities with greater match involvement (on-field)	On-field referees reported higher levels of psychological distress compared with line referees
Brick et al. [6]	Cross-sectional	Prevalence and frequency of verbal and physical abuse, psychological distress, mental health outcomes and intentions to quit	438 (4:434) *M*_age_ 45.2 ± 11.1 Ireland	Various Gaelic sports Club—National	Age Years of officiating experience Perceptions of adequate mental health training Experiences of Abuse when officiating (Physical and Verbal) questionnaire	Distress Screener WEMWS (short form) PHQ-2 GAD-2	Older Age More years of officiating experience	Lower years of officiating experience Frequency of verbal abuse Perceived lack of adequate mental health training Experiences of physical abuse	**Prevalence:** 5.25% and 5.48% of participants screened positive for anxiety and depression respectively Older referees’ higher mental well-being, lower anxiety and lower depression Frequent verbal abuse associated with higher symptoms of depression, anxiety and stress, and lower mental well-being Feelings of distress from physical abuse correlated with lower mental well-being, higher anxiety, higher depression Perceived inadequacy of training associated with higher distress after verbal abuse, lower mental well-being, higher anxiety, higher depression
Carson et al. [29]	Cross-sectional	Explore mental health, officials' psychological well-being and the individual and workplace factors associated with these states	317 (73:244) Age range: 18–30 (20%), 31–50 (36%), > 50 (43.8%) Australia	Various sports Local/community –International	Age Sex Years of officiating experience Level of workload manageability and control Relationship status Level of formal education	AWS DASS-21 WEMWS	Sex (Male) Older age (> 50 years) More years of officiating experience Greater workload manageability and workload control	Younger age (18–30 years) Relationship status (single) Low level of formal education (not attended university) Lower years of officiating experience	Males, who were older (> 50 years), married/committed, and had higher education showed higher levels of psychological well-being than their female counterparts Being older (> 50 years), having greater officiating experience (6–9 years, ≥ 10 years), workload manageability and workload control were associated with lower DASS-21 scores Single/divorced/separated/widowed and having a high school education associated with lower DASS-21, lower well-being
Dorsch and Paskevich [30]	Cross-sectional	Explore intensity of perceived stressful situations among the six certification levels of Canadian ice hockey officials	421 (13:408) *M*_age_ 26.2 ± 11.42 Canada	Ice Hockey Level 1—Level 6^a^	Officiating level Verbal and physical abuse Fear of mistakes	Hockey Officials' Sources of Stress Inventory	Officiating in lower levels of competition (level 1)^a^		Intensity of feelings of stress from verbal and physical abuse differed across certification levels Level 1 officials perceived significantly less stress from abusive events than levels 2–6
Elsworthy et al. [31]	Observational	Examine the effect of match schedule on self-reported wellness and sleep in rugby union referees during the 2019 Rugby World Cup	18 (All male) Multiple countries	Rugby Union International	Density of match scheduling	Daily wellness status questionnaire		Density of match scheduling (single-game regular match schedules)	Significant reduction in all self-reported wellness variables except stress during regular match schedules Single-game regular match schedules had a negative impact on sleep
Fernandez et al. [32]	Cross-sectional	Explore relationship with emotional intelligence, burnout, and perception of health in soccer referees	4099 (324:3775) Age range: 14–66 years Spain	Soccer Amateur—Professional	TMMS-24	GHQ-12 OLBI	High emotional intelligence in the clarity and repair dimensions More years of officiating experience	Low emotional intelligence in the attention dimension	Attention dimension of EI was positively associated with distress. Clarity dimension and repair dimension of EI had a negative association with distress Negative correlation between years of experience and burnout scores
Gama et al. [33]	Cross-sectional	Explore burnout in amateur and professional soccer referees	36 (4:32) M_age_ 32.2 ± 5.6 Brazil	Soccer 52.7% Amateur (47%) and Professional (53%)	Level of competition officiated Years of officiating experience	BIR		Officiating in lower levels of competition (Amateur) Lower years of officiating experience	Amateur league referees showed significantly higher levels in the sport devaluation component of the BIR than professional league referees In amateur referees, refereeing time was positively associated with the BIR's reduced sense of sports accomplishment sub-scale
Gorczynski and Thelwell [34]	Cross-sectional	Explore levels of mental health literacy, depressive symptoms, well-being and help-seeking behaviours of soccer match officials	313 (45:268) M_age_ 27.4 ± 8.7 UK	Soccer 42.5% Level 7	Sex Level of competition officiated Hours of officiating per week MHLS GHSQ (one question)	CESD-R WEMWBS	Less hours officiating per week (1–3 h) Sex (Male) Higher levels of mental health literacy Higher levels of help-seeking intentions	Officiating in lower levels of competition Sex (Female)	CESD-R scores revealed 2% of referees indicated symptoms for major depressive episodes, 7% for a probable depressive episode, 2% for a possible depressive episode, and 88% scored subthreshold (≥ 16) Female referees higher CESD-R scores than males CESD-R scores lower when officiating 1–3 h per week GHSQ scores higher when officiating 1–3 h per week than 4 or more hours per week WEMWBS scores higher for males relative to females
Gouttebarge et al. [35]	Prevalence (observational prospective cohort study)	Prevalence and incidence of symptoms of common mental disorders	391 (NR) M_age_ 33 ± 7 Multiple countries	Soccer Professional		Distress screener GHQ-12 PROMIS (short form) Eating disorder screen for primary care AUDIT-C View on consequences, support, and needs			**Prevalence:** Baseline (4-week) prevalence of symptoms was 6% for distress, 12% for anxiety/depression, 9% for sleep disturbance, 19% for eating disorders and 17% for adverse alcohol use The one-season incidence of symptoms was 10% for distress, 16% for anxiety/depression, 14% for sleep disturbance, 29% for eating disorders and 8% for adverse alcohol use 18% of referees sought medical help for symptoms 70% stated the need for specific mental health support measures for referees to be developed
Johansen and Haugen [36]	Cross-sectional	Explore levels of anxiety and its impact on officiating	98 (10:88) *M*_age_ 33.2 ± 7.2 Norway	Soccer Second League—Premier League	Level of competition officiated Perceived refereeing competence Perceived social pressure, noise disturbance and aggressive behaviour from coaches, players and substitutes (nine statements total)	STAI-Y	High levels of perceived role competence	Officiating in higher levels of competition Experiences of abuse (aggressive behaviour from players or coaches)	Referees adjudicating the highest division higher levels of anxiety than second division Referees who perceived competence as being the 'best' or ‘very best’ had lower STAI-Y
Karaçam et al. [37]	Cross-sectional	Examine mental well-being and emotional regulation in basketball referees	327 (44:283) *M*_age_ 28.7 years Turkey	Basketball Regional—National	Age Level of competition officiated Years of officiating experience	WEMWBS RERS	Older age More years officiating experience Officiating in higher levels of competition (National)	Officiating in lower levels of competition (Regional)	WEMWBS correlated with age and years of experience. Nationally accredited referees higher WEMWBS relative to regional referees
Kilic et al. [38]	Prevalence (observational prospective cohort study)	Explore the association of physical and psychosocial stressors with symptoms of common mental disorders	391 (NR) *M*_age_ 33 ± 7 Multiple countries	Soccer Professional	Satisfaction with social/organisational support History of severe injury	Distress screener GHQ-12 PROMIS (short form) Eating disorder screen for primary care AUDIT-C		Reduced satisfaction with social/organisational support History of severe injury (higher number of injuries)	**Prevalence:** Baseline (4-week) prevalence of symptoms was 5.9% for distress, 11.8% for anxiety/depression, 9.1% for sleep disturbance, 19.2% for eating disorders and 16.5% for problematic alcohol misuse Referees exposed to severe injuries and/or exhibit a lower satisfaction of social support were more likely to report symptoms of anxiety/depression Referees reporting low levels of satisfaction with social support were more likely to report symptoms of eating disorders
Kim et al. [39]	Cross-sectional	Explore relationships between referee retention, authenticity at work, referee engagement, and psychological well-being	410 (22:388) *M*_age_ 48.6 ± 12.3 US	Basketball Varsity-level high (88%) and College/ Professional (28%)	Job engagement scale-modified Authenticity Measure at Work	FS	Level of referee engagement Level of authenticity at work		Referee engagement predictor of FS scores Authenticity at work positive relationship with FS scores
Koh and Hanrahan [40]	Cross-sectional	Explore the role of individualism-collectivism continuum, in relation to basic psychological needs, motivation and burnout in netball umpires	72 (All female) *M*_age_ 34 ± 12.2 Multiple countries	Netball International	BNS Modified Individualism and Collectivism Scale Behavioural regulation in sport questionnaire	ABQ MBI			Multivariate effect of individualism across BNS, ABQ and MBI
Lima et al. [41]	Prevalence (cross-sectional)	Prevalence of Mental health problems and association with demographic variables in Turkish amateur football league referees	1279 (59:1220) Turkey	Soccer Level not specified	Sex Age Relationship status Annual income Years of officiating experience History of severe injury Concerns about performance Satisfaction with social/organisational support	PHQ-9 GAD-7 PSS-10		Sex (Female) Younger age Relationship status (single) Low annual income Lower years of officiating experience History of severe injury (Higher number) Concerns about performance Reduced satisfaction with social/organisational support	**Prevalence:** 7.1% and 3.8% reported moderate to severe symptoms of depression 4.8% and 1.1% reported moderate-to-severe symptoms of anxiety 2.4% and 42.8% reported moderate-to-severe symptoms of stress Females reported higher levels of depression and anxiety relative to males Anxiety and depressive symptoms higher in younger referees (38.2%) compared to older referees (6.6%) Single referees higher symptoms depression, anxiety, and stress Lower income associated with higher symptoms depression, anxiety, and stress Referees with less refereeing experience (4–7 years) higher anxiety scores than more experienced officials (12–15 years) Referees with a severe sports injury history, performance concerns, and perceived inadequate social support reported higher depression, anxiety, and stress
Lima et al. [42]	Prevalence (cross-sectional)	Prevalence of Mental health problems and association with demographic variables in Turkish amateur football league referees	433 (5:428) *M*_age_ 33 ± 7 Turkey	Soccer Professional	Age Level of competition officiating Level of formal education Years of officiating experience Annual income Relationship status Concerns about performance History of severe injury Satisfaction with social/organisational support	PHQ-9 GAD-7 PSS-10	Higher levels of formal education More years officiating experience	Younger Age (18–27 years) Officiating in lower levels of competition Low annual income Relationship status (single) Concerns about performance History of severe injury (higher number of injuries) Reduced satisfaction with social/organisational support	**Prevalence:** PHQ-9: Mild (24.5%), Moderate (7.6%), Severe (2.5%) GAD-7: Mild (18.7%), Moderate (4.4%), Severe (0.9%) PSS-10: Moderate (38.8%), Severe (1.8%) Younger referees (18–27 years) higher symptoms of depression, anxiety, and stress than older referees (> 38 years) Low-level referees (C class) higher depression, anxiety, and stress compared to higher-level (Super League) referees Referees with ≥ 15 years of experience lower stress compared to 4–7 years officiating experience Depression, anxiety, and stress higher for referees with an annual income of $5000 ≤ compared with those earning 30,000 ≥ annually Single referees with performance concerns, a history of severe injury, and perceived social support to be inadequate reported higher depression, anxiety, and stress
Liu et al. [43]	Prevalence (cross-sectional)	Investigate relationship between stress and mental health and the moderating role of perceived social support	317 (146:171) 19–45 years China	Soccer Level not specified	Age Perceptive Social Support Scale ERI (Occupational stress measure)	DASS-21 MBI-GS		Increased levels of occupational stress Younger age	**Prevalence:** Mild or above symptoms of depression (16.1%), anxiety (22.7%) and stress (5.9%) Occupational stress was negatively correlated with perceived social support, but positively correlated with, depression, and anxiety Job burnout was positively correlated with depression, anxiety Age significantly correlated anxiety
Lishman et al. [44]	Prevalence (cross-sectional)	Determine impact of abuse and other stressors on mental health outcomes	303 (2:301) *M*_age_ 45.19 ± 12.28 Ireland	Various Gaelic sports Club—National	RSS RCS Brief Cope (Social media abuse coping)	Distress Screener MHC-SF PHQ-8 GAD-7		Higher frequency of verbal abuse Greater use of avoidance-cognitive coping	**Prevalence:** 17.8% reported mild, 5.28% moderate, 0.99% moderately severe and 0.66% severe depression. 16.50% reported symptoms of mild, 5.61% moderate and 2.31% severe anxiety. Mental well-being data indicated that 69.6% were classified as flourishing, 27.7% as moderately healthy and 2.8% as languishing Experiencing more frequent verbal abuse associated with higher distress, higher anxiety, higher depression and lower total well-being Greater use of avoidance-cognitive coping was associated with higher distress after verbal abuse, higher anxiety, higher depression
Martínez-Moreno et al. [45]	Cross-sectional, observational study	Explore the relationship of leadership style with burnout level, emotional exhaustion, reduced personal accomplishment and depersonalisation in basketball referees	61 (9:52) 19–55 years No country specified	Basketball Local—National	Multifactor Leadership Questionnaire Reviewed Referee responsibility (Main or on-field) Level of competition officiating	Athlete Burnout Inventory Stress scale for sports	Transformational and developer leadership styles	Passive leadership style Responsibilities with greater match involvement (Main, on field referee) Officiating in lower levels of competition	Transformational and developer styles were most used and associated with lower levels of stress and high levels of personal accomplishment The passive leadership style is associated with high levels of stress and low levels of personal accomplishment Higher levels of stress and burnout based on referee role (main, assistant, other) level of officiating (local, regional, national)
Nesti and Sewell [46]	Longitudinal observational study with both qualitative and quantitative components	Explore how mood and anxiety fluctuated in response to significant life events, both sport-related and non-sport-related	15 (NR) 19–55 years UK	Netball and Rugby League Professional Local—National		Likert-type scale for mood and anxiety Sleep Journal		Reduced sleep quality	Significant correlation for all subjects between weariness and workload
Onturk et al. [47]	Cross-sectional	Examine the level of anxiety of the referees who are active in different regions of refereeing	167 (40:127) Turkey	Sport not specified Various levels	Gender	STAI—adapted		Sex (Female)	Female referees higher trait anxiety levels compared to male referees
Orviz-Martínez et al. [48]	Cross-sectional	Explore whether the environment and violence influence burnout in a sample of grassroots football referees	203 (12:191) 23.5 years Spain	Soccer Amateur	Cynicism; Effectiveness Sense Verbal or physical aggression Influence of Environment	MBI-GS Emotional Exhaustion		Experiences of verbal and physical aggression Pre-existing knowledge of match environment (Known players, recording medium) Increased levels of cynicism	Environment around matches and the level of verbal and physical aggression experienced by referees associated with increased emotional exhaustion and cynicism and decreased their sense of professional effectiveness ‘Vicious cycle’ was identified as Verbal or physical aggression and influence of the environment (initiators) > emotional exhaustion > professional efficiency > cynicism > emotional exhaustion
Stewart and Ellery [49]	Cross-sectional	Assess whether high-school volleyball coaches they reported a magnitude of stress similar to that of officials in previous research	353 (222:131) *M*_age_ 37.8 ± 9.2 US	Volleyball High-school level		Self-reported psychological stress			No significant findings
Taylor et al. [50]	Longitudinal observational study with both cross-sectional and longitudinal analyses	Investigate the role of burnout as a mediating affective response between perceived stress and dropout intentions among soccer officials over the course of a soccer season	529 (NR) *M*_age_ 38.7 ± 11 Canada	Soccer Amateur—National	Age Level of competition officiating Fear of failure Perceived stress from interpersonal conflicts Role-culture conflict	MBI—modified Ontario Soccer Officials survey		Older age Officiating in higher levels of competition officiated Fear of failure Perceived stress from interpersonal conflicts Conflicts arising from misalignment between officials' expectations for recognition and officiating environment (i.e., role-culture)	Older officials have lower levels of burnout Fear of failure was the strongest predictor of burnout, followed by interpersonal conflicts, and role-culture conflicts
Wang et al. [51]	Cross-sectional	Explore the impact of the COVID-19 lockdown on the quality of life of Chinese football referees, as well as the influence of occupational stress and job burnout	307 (52:255) 19–45 years China	Soccer Level 3 – National	Impact of Event Scale (COVID-19) ERI –Revised (Occupational stress)	MBI-GS WHOQOL-BREF		Environmental restrictions around officiating activities (e.g., COVID-19) Increased levels of occupational stress	COVID-19 isolation was positively correlated with occupational stress and job burnout and negatively correlated with quality of life Occupational stress and job burnout were positively correlated with quality of life

*ABQ* Athlete Burnout questionnaire, *AWS* Areas of work life Scale, *AUDIT-C* alcohol use disorders identification test, *BIR* Burnout Inventory for Referees, *CESD-R* Centre for Epidemiologic Studies Depression Scale-Revised, *COPQQ* Copenhagen Psychosocial Questionnaire, *DASS-21* Depression Anxiety and Stress Scale 21, *ERI* Effort Reward Imbalance Scale, *FS* Flourishing Scale, *GAD-7* Generalised Anxiety Disorder-7, *MBI* Maslach Burnout Inventory, *MBI-GS* Maslach Burnout Inventory-General Survey, *MHLS* Mental Health Literacy Scale, *OLBI* Oldenburg Burnout Inventory, *PROMIS* Patient-Reported Outcomes Measurement Information System, *PHQ-9* Patient Health Questionnaire-9, *PSS-10* Perceived Stress Scale-10, *RERS* Referee Emotion Regulation Scale, *STAI-Y* State-Trait Anxiety Inventory, *TMMS-24* Trait Meta-Mood Scale-24, *WEMWS* Warwick-Edinburgh Mental Wellbeing Scale, *WHOQOL-BREF* World Health Organisation Quality of Life Brief Version

^a^The six certification levels of Canadian Ice Hockey Officials referenced in Dorsch & Paskevich [30] are defined as follows: level 1: entry-level, involving officiating youth and minor hockey; level 2: officiating of high levels within minor hockey; level 3: officiating of competitive youth leagues and recreational adult hockey, expected to demonstrate advanced rule knowledge; level 4: officials with significant experience and capable of officiating high-level junior hockey and provincial championships; level 5: qualified to officiate at highest levels of junior hockey, senior leagues, and national championships; level 6: national and international officiating, including professional leagues and international tournaments

**Table 2:**
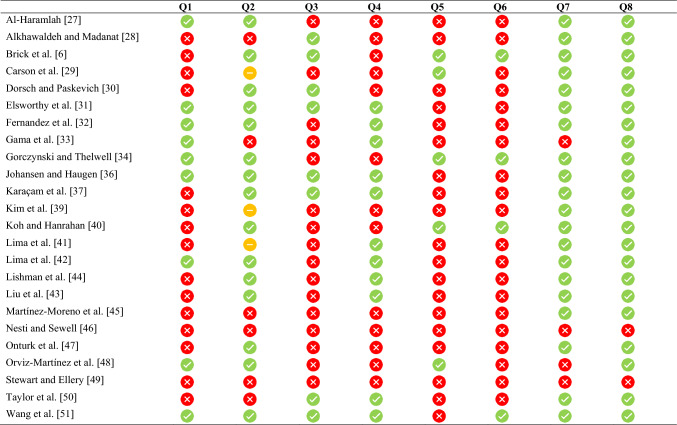
Quality Assessment of cross-sectional studies

Assessed using the JBI critical appraisal checklist for analytical cross-sectional studies. JBI, Joanna Briggs Ins. Green ticks represent yes, red cross represents no and orange dash represents other/not reported

**Table 3:**
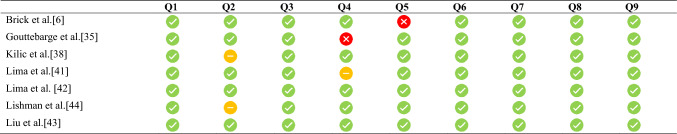
Quality Assessment for studies reporting mental health symptom prevalence

Assessed using the JBI critical appraisal checklist for prevalence data. JBI, Joanna Briggs Institute. Green ticks represent yes, red cross represents no and orange dash represents other/not reported

### Prevalence and Incidence of Mental Health Symptoms and Disorders

Seven studies (27%) reported prevalence rates of mental health symptoms (e.g. anxiety and depression). Two studies were longitudinal (e.g. prospective cohort design [35, 38]), with the remaining five studies being cross-sectional designs. All seven studies reporting prevalence data assessed mental health symptoms/disorders using validated self-report measures (e.g. Patient Health Questionnaire) to quantify symptoms of mental disorders [6, 35, 38, 41–43]. None of these studies reported the use of a diagnostic interview to verify symptoms and provide a formal diagnosis of a mental health disorder (e.g. major depressive disorder). Studied mental health symptoms included: depression [6, 41–43], anxiety [6, 41–43], sleep-disturbance [35, 38], eating disorder symptoms [35, 38] and symptoms of problematic alcohol use [35, 38]. The prevalence of these symptoms was reported primarily in soccer officials (*k* = 5) performing at a sub-elite to elite level. The reported proportion of elevated depression symptoms in each sample varied from 5.3% [6] to 36.1% [42]. Regarding symptoms of anxiety, the reported proportion varied from 5.5% [6] to 26.2% [42]. Two studies reported prevalence rates from the same data set for sleep disturbance (9.1%), eating disorders (19.2%) and problematic alcohol use (16.5%) [35, 38].

**Fig. 2:**
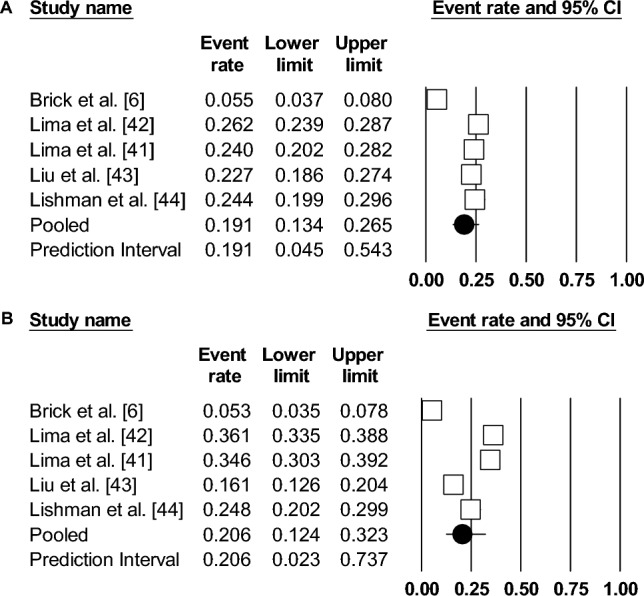
Forest plots showing proportions of anxiety and depression symptoms in *k* = 5 studies. **A** Symptoms of Anxiety; **B** symptoms of depression. The pooled effect is denoted by the circle. Squares denote the proportion of anxiety/depression symptoms for each individual study and the horizontal lines denote the 95% CI for each individual study

From a cumulative sample of *N* = 2797 officials (*k* = 5, median *n* = 433), the pooled proportion of elevated anxiety symptoms was 19.1% (95% confidence interval [CI] 13.4–27, *I*^2^ = 94.1%) (Fig. 2). Further analyses showed no evidence of publication bias (Kendall’s *t*, *Z* = 1.22, *p* = 0.11; Egger’s test, *t*(3) = 2.09, *p* = 0.06). Duval and Tweedie’s trim and fill method (random effects model) imputed no effect sizes to the left/right of the mean, leaving the pooled prevalence rate unchanged (Supplementary Fig. S1). The pooled proportion of elevated depression symptoms was 20.6% (95% CI 12.4–32.3, *I*^2^ = 97.3%) in a cumulative sample *N* = 2797 officials (*k* = 5, median *n* = 433). Further analyses suggested evidence of publication bias (Kendall’s *t*, *Z* = 2.20, *p* = 0.01; Egger’s test, *t*(3) = 3.83, *p* = 0.01). Under Duval and Tweedie’s trim and fill method (random effects model), no effect sizes were imputed, leaving the pooled prevalence rate unchanged (Supplementary Fig. S1). Unfortunately, the number of unique datasets was too small for further subgroup analyses.

Incidence rates were reported in one of the included studies [35]. One-season incidence of mental health symptoms for elite soccer officials was 14% for sleep disturbance, and 8% for problematic alcohol use. From this study, an incidence rate of 29% was observed for symptoms of eating disorders, assessed by a broad eating disorder screening tool.

#### Prevalence and Incidence of Psychological Distress

The prevalence of psychological distress was reported in only one study [35], with a prevalence rate of 6%. Brick et al. [6] was unique from other studies, in that psychological distress was reported only for those in their sample that had experienced abuse. Prevalence rates of those who screened positive for distress during the week after receiving verbal and physical abuse were reported as 5.08% and 12.78% respectively. One-season incidence of psychological distress for elite soccer officials was 10%. One dataset reported across two studies assessed and reported combined symptoms of depression and anxiety [35, 38], reflecting an approach that conceptualises these co-occurring symptoms as indicators of overall psychological distress. Subsequently, owing to the variation and ambiguity of included studies regarding psychological distress, meta-analysis was deemed inappropriate on this variable.

### Risk and Protective Factors

#### Factors Affecting Officials’ Mental Health

Of the included studies, we identified 47 correlates related to sporting officials’ mental health. These variables were organised into 21 broad themes following extraction (13 risk factors and eight protective factors; see Table 4). Sport-environmental risk factors were investigated in 69% of the included studies (*k* = 18) (e.g. levels of professional experience, environment around matches, experiences of abuse), while 50% of studies (*k* = 13) examined personal risk factors (e.g. age, sex, injury). A total of 38% of studies (*k* = 10) examined sport-environmental protective factors (e.g. years of officiating experience, level of officiating, hours and frequency of officiating), while 35% of studies (*k* = 9) investigated personal factors (e.g. emotional intelligence, age, sex). The relationship between level of officiating experience (e.g. officiating higher/lower levels of competition, more/less years of officiating experience) and officials’ mental health was the most researched risk/protective factor theme, assessed in 65% of studies.

**Table 4:** Protective and risk factors influencing officials’ mental health

Factors			References	*n*	%
Protective/risk factors	Individual factors	Associated mental health outcomes			
Personal protective factors					
Age (older)	–	↓Symptoms of anxiety and depression ↑Mental well-being ↑Work control and manageability	[6, 29, 37, 41]	4	15
Gender (Male)	–	↑Psychological/mental well-being ↓Symptoms of depression and anxiety ↓likelihood of quitting officiating	[29, 34, 42, 47]	4	15
Emotional and cognitive resources	High emotional intelligence in the clarity and repair dimensions Transformational and developer leadership styles Higher levels of formal education Higher levels of perceived role competence	↑Perceived overall health ↓Levels of distress ↓Symptoms of depression, anxiety and stress ↑Levels of perceived personal accomplishment ↑Levels of perceived role competence	[32, 36, 41, 45]	4	15
Mental health literacy	Higher levels of mental health literacy	↓Symptoms of depression ↑Mental well-being ↑Intentions to seek mental health help (informal and formal sources)	[34]	1	4
Help-seeking intentions	Higher levels of help-seeking intentions	↓Symptoms of depression ↑Mental well-being	[34]	1	4
Sport-Environmental Protective Factors					
Level of officiating experience	Less hours officiating per week Higher number of games officiated in previous season More years officiating experience Higher level of competition officiated Lower level of competition officiated	↓Symptoms of depression and burnout ↑Mental well-being ↑Ability to emotionally regulate	[6, 29, 30, 32, 34, 37, 41]	7	27
Positive workplace engagement	Level of referee engagement Level of authenticity at work	↑Psychological well-being	[39]	1	4
Effective workload management	Workload manageability Workload control	↓Symptoms of anxiety, depression and stress	[29]	1	4
Personal Risk Factors					
Age (younger)	–	↑Symptoms of depression, anxiety, stress, and burnout	[29, 41–43, 50]	5	19
Gender (Female)	–	↑Symptoms of depression and anxiety ↓Psychological/mental wellbeing	[29, 34, 42, 47]	4	15
History of severe injury	–	↑Symptoms of depression, anxiety and stress	[38, 41, 42]	3	12
Socioeconomic factors	Low annual income Relationship status (single) Full-time employment (in addition to officiating duties) Low level of formal education (not attended university)	↑Symptoms of depression, anxiety and stress ↓Mental well-being	[29, 41, 42]	3	12
Cognitive and emotional factors	Increased levels of cynicism Low emotional intelligence in the attention dimension Passive leadership style Avoidance Coping Style	↑Symptoms of depression, anxiety ↑Emotional exhaustion ↑Symptoms of burnout and stress ↑Perceived poor health ↓Perceived personal accomplishment	[32, 44, 45, 48]	4	15
Negative cognitive content about performance	Concerns about performance Thinking about past failures Fear of failure	↑Symptoms of depression, anxiety, stress and burnout ↓Desire to continue officiating	[36, 41, 42, 50]	4	15
Sport-Environmental risk factors					
Preparation for competition	Reduced sleep quality Perceived lack of adequate mental health training Density of match scheduling	↑Levels of distress ↓Self-reported wellness ↑Symptoms of anxiety	[6, 31, 46]	3	12
Level of officiating experience	Lower years of officiating experience Officiating in lower levels of competition Officiating in higher levels of competition	↑Symptoms of depression, anxiety, stress and burnout	[6, 29, 33, 34, 36, 37, 41, 42, 45, 50]	10	34
Environmental factors	Pre-existing knowledge of match environment Environmental restrictions around officiating activities (e.g., COVID-19)	↑Emotional exhaustion ↑Cynicism ↑Levels of occupational stress ↑Symptoms of burnout ↓Quality of life ↓Sense of professional effectiveness	[48, 51]	2	8
Workplace stress and pressure	Increased psychological pressure from in-match decision-making Increased levels of occupational stress	↑Frequency and intensity of burnout symptoms ↑Symptoms of depression and anxiety ↑Levels of pressure experienced	[27, 43, 51]	3	12
Role responsibilities	Responsibilities with greater match involvement	↑Levels of burnout	[28, 45]	2	8
Experiences of verbal and/or physical abuse, and/or social media abuse	–	↑Symptoms of anxiety, depression and stress ↓Mental well-being	[6, 36, 44, 48]	4	15
Organisational issues	Reduced satisfaction with social/organisational support Perceived lack of social support Conflicts arising from misalignment between officials' expectations for recognition and officiating environment (i.e., role-culture) Perceived stress from interpersonal conflicts	↑Levels of burnout ↑Symptoms of anxiety, depression and stress ↑Symptoms of eating disorders (broadly defined)	[38, 41, 42, 50]	4	15

#### Personal Protective Factors

Older age was found in four studies to be associated with improved mental health outcomes, such as lower levels of anxiety and depression symptoms, increased mental well-being and greater work control and manageability [6, 29, 37, 41]. Four studies found that male gender was a protective factor, with males reporting lower symptoms of depression, anxiety and higher levels of psychological well-being [29, 34, 42, 47] compared with their female counterparts. Emotional and cognitive resources were investigated in four studies. Specifically, two dimensions of emotional intelligence were related to an improved perception of overall health, including officials’ ability to understand and accurately identify one’s emotions (clarity dimension), and the capacity to effectively regulate and manage negative emotions (repair dimension) [32]. Transformational and developer leadership styles, which focus on inspiring and motivating others while fostering personal growth and skill development, were associated with reduced levels of distress and higher levels of perceived personal accomplishment [45]. Officials with higher levels of formal education (e.g. university versus high school) reported significantly lower symptoms of depression and distress relative to their counterparts [41]. Officials with high levels of perceived role competence—the confidence in their ability to perform their duties effectively—reported lower levels of anxiety symptoms, suggesting that their heightened sense of competence may be related to a reduction in symptoms of anxiety [36]. Mental health literacy and help-seeking intentions were identified as protective mental health factors in one study [34]. Higher levels of mental health literacy—the knowledge and understanding of poor mental health and available treatments—were found to be associated with lower symptoms of depression, higher levels of mental well-being and greater intentions to seek mental health help. This increased intention to seek help (both formal and informal sources of help) for mental health was linked with increased levels of mental well-being and reduced levels of depression.

#### Sport-Environmental Protective Factors

Seven studies highlighted the positive influence of the ‘level of officiating experience’ on officials’ mental health: specifically, fewer hours of officiating per week [34], more years of officiating experience [6, 29, 32, 37, 41] and higher level of competition officiated (e.g. elite) [32, 37]. These factors were found to be positively associated with officials' mental health, including lower symptoms of depression, increased mental well-being, lower symptoms of burnout and increased ability to emotionally regulate. It is important to consider the nuances of the officiating role when interpreting these results, as a more experienced official may choose to officiate less often, whereas a less experienced official may choose to officiate more often. While these studies provide some insight into the role of experience on officials’ mental health, future work could explore this in more detail such as through qualitative and/or case study methodologies. Interestingly, one study revealed contrary findings, showing that individuals officiating in lower-level competition perceived significantly lower levels of distress from sport-specific stressors (e.g. fear of mistakes) compared with higher-level officials [30]. One study examined ‘positive workplace engagement’ factors, specifically referee engagement—defined as the affective-motivational state of work-related well-being—and authenticity at work, or the capacity to express one’s true self at work [39]. Findings revealed that both factors were linked to increased psychological well-being. ‘Effective workload management', specifically, an official’s ability to control and manage their officiating load, was investigated in one study and was associated with fewer symptoms of anxiety, depression and stress [29].

#### Personal Risk Factors

Five studies found that younger age had a negative association with officials’ mental health, including higher symptoms of depression, anxiety, stress and burnout [29, 41–43, 50]. In four studies, female officials were found to be at higher risk of heightened symptoms of depression, anxiety and significantly lower levels of psychological well-being, relative to males [29, 34, 42, 47]***,*** though female officials only comprised 10% of participants across these studies. Reported ‘history of severe injury’ was found to be a factor linked with adverse mental health outcomes in three studies, included higher levels of symptoms of psychological distress (e.g. anxiety, depression, stress) [38, 41, 42]. ‘Socioeconomic factors’ were associated with poor mental health outcomes in four of the included studies. Specifically*,* low annual income [41, 42], being single [41, 42], being in full-time employment (in addition to officiating duties [42]) and having a low level of formal education (e.g. not attended university) [29], were associated with higher levels of psychological distress (e.g., anxiety, depression and stress), and reduced mental well-being. ‘Cognitive and emotional factors’ were identified in four studies as being associated with poorer mental health including cynicism [48], low emotional intelligence in the attention dimension (the tendency to fixate excessively on one’s own emotions [32]), avoidance-cognitive coping style [44] and a passive leadership style (characterised by inaction or avoidance in guiding others) [45]. Specifically, these emotional and cognitive factors were linked to increased symptoms of depression, anxiety, burnout and elevated levels of psychological distress. ‘Negative cognitive content about performance’ was investigated in four studies, including concerns about performance [41, 42], thinking about past failures [36] and fear of failure [50]. These factors were found to be associated with higher reported symptoms of depression, anxiety, stress and burnout.

#### Sport-Environmental Risk Factors

‘Preparation for competition’ factors were identified in three studies, including reduced sleep quality [46], perceived lack of adequate mental health training [6] and the density of match scheduling [31]. These factors were found to be associated with increased levels of distress, reduced self-reported well-being and higher symptoms of anxiety. Ten studies showed that ‘level of officiating experience’ was negatively associated with officials' mental health. Specifically, studies showed factors such as lower years of officiating experience [6, 29, 42] and officiating in lower levels of competition (e.g. amateur) [33, 34, 37, 41, 42, 45, 50] were related to higher symptoms of anxiety, depression, stress and burnout. Interestingly, one study found that referees officiating in higher levels of competition had significantly greater levels of anxiety relative to their less skilled counterparts [36]. ‘Environmental factors’ were examined in two studies, including pre-existing knowledge of match environment (i.e. prior knowledge of certain teams and/or players when officiating [48]) and environmental restrictions around officiating activities (i.e. COVID-19 [51]). Such factors were found to be associated with poor mental health outcomes in officials, including higher symptoms of burnout and reduced psychological well-being (e.g. reduced quality of life). Three studies identified increased levels of ‘stress and pressure’ as risk factors*,* including pressure from in-match decision-making [27] and increased levels of occupational stress [43, 51]. These factors were linked with adverse outcomes such as increased frequency and intensity of burnout symptoms, and increased symptoms of depression and anxiety. Officials who carried out ‘role responsibilities’ that required greater match involvement (e.g. on-field versus assistant referee) were found to experience higher levels of burnout [27, 45]. ‘Experiences of verbal and/or physical, and/or social media abuse’ were investigated in four studies [6, 36, 44, 48] and were found to have a strong negative impact on mental health outcomes, including increased psychological distress, reduced psychological well-being and higher levels of anxiety and depression. ‘Organisational issues’ were identified as risk factors in four studies. Specifically, three studies reported reduced satisfaction with social/organisational support to be associated with increased symptoms of anxiety, depression, stress and eating disorders (broadly defined) [38, 41, 42]. The remaining study revealed that misalignment between officials' expectations for recognition and officiating environment (i.e. role-culture), and perceived stress from interpersonal conflicts were associated with higher levels of burnout among officials [50]. It is important to note that in this study, the construct of interpersonal conflict was not defined; therefore, it is unclear whether this refers to conflict between players, coaches or other staff members within an official’s organisation.

## Discussion

Although there has been an increased focus on athletes’ mental health in recent years, our understanding of the impact of unique stressors on sporting officials is limited. This review examined the literature to identify current estimates of mental health symptoms and disorders prevalence among sporting officials. In addition, this review summarises currently known risk and protective factors that influence officials’ mental health symptoms, disorders and/or psychological well-being. As Webb et al. [14] identified in their recent expert statement on sport officiating research, little is known about how to best protect and promote mental health in officiating, and this review assists to collate the existing knowledge on this topic. Our meta-analysis showed that the prevalence of elevated mental health symptoms was 19.1% for anxiety and 20.6% for depression in a cumulative sample of 2797 sporting officials. Psychological distress was investigated in only one study, reporting a prevalence rate of 6%. The systematic review identified key risk factors associated with poor mental health outcomes, including younger age and fewer years of officiating experience. These factors were linked to higher levels of depression, anxiety, stress and burnout symptoms. Factors such as more years of officiating experience and older age were identified as being protective, linked with increased levels of psychological well-being and decreased symptoms of depression, anxiety and stress.

Our findings suggest that a large proportion of sporting officials may be experiencing symptoms of psychological distress, specifically anxiety and depression symptoms. The pooled prevalence rate for anxiety (19.1%) and depression symptoms (20.6%) was found to be lower than in other sporting populations, such as current and former elite athletes [1]. However, in a recent systematic review and meta-analysis performed across various sports and nations, the pooled prevalence rate of distress reported anxiety and/or depression symptoms combined (34% [1]), indicating that the individual rates of depression and anxiety reported in our review may potentially be higher than previously indicated rates in athletes. Alternatively, in reporting rates separately for anxiety and depression our results might also reflect comorbidity of depression and anxiety symptoms. Clinically, affective disorders, such as depression or anxiety disorders have distinct symptomology. For example, depression is characterised by low mood, with common symptoms including amotivation and anhedonia, whereas anxiety disorders are characterised by worry or fear. Therefore, it is recommended that for future studies on mental health in sport that researchers aim to report mental health symptoms separately to further our understanding of the prevalence of various mental health symptomologies.

More years of officiating experience and higher emotional and cognitive resources (e.g. high levels of formal education) were consistently identified as fostering improved mental health outcomes, such as reduced distress and increased perceived role competence. A potential explanation for this is that more years of experience may facilitate officials in developing the necessary emotional and cognitive resources to successfully navigate the pressures of their roles more effectively and mitigate potential negative mental health outcomes [32, 36, 41, 42, 45]. The emergence of higher emotional intelligence in the clarity and repair dimensions (i.e. the capacity to regulate and manage negative emotions effectively) as a protective factor provides partial support for this idea [32]. Officials with more years of experience may have also developed adaptive thought patterns that enable them to reframe challenges, manage cognitive stressors and sustain a sense of competence in high-pressure environments. This may reflect a gradual process of refinement of more experienced officials' cognitive and emotional skills through repeated exposure to stressors and the development of successful coping strategies [37, 41].

In contrast to the positive mental health outcomes predominantly reported for older and/or more experienced officials, our results highlight that younger officials and/or those with lower levels of experience (e.g. lower years and levels of competition) are at heightened risk of experiencing poor mental health outcomes, including higher levels of depression, anxiety and psychological distress [29, 41–43, 50]. These findings may partly reflect the higher rates of poor mental health that emerge in young adulthood within the general population [52]. However, evidence from the present review suggests that mental-health-related attrition may also play a significant role, with younger, less experienced officials experiencing poor mental health being more likely to discontinue officiating [6, 36]. By contrast, older, more experienced officials with positive mental health may remain in their roles, contributing to the evident differences in mental health outcomes across age and experience levels. Concerningly, our review highlights that very little is known about mental health outcomes among young officials, with a notable lack of research on sporting officials under 18 years of age. Retaining young officials is crucial to the sustainability of organised sports, with research indicating an increased demand for sporting officials, highlighted by substantial declines in the participation of sporting officials worldwide [53]. Further investigation is needed to understand how best to support young officials in the early stages of their careers and implement early intervention frameworks that provide targeted interventions to improve mental health outcomes.

Our findings support the proposition of Webb et al. [54], who suggested that the impact of abuse on sporting officials should be viewed as a public health issue. Our results identified abuse as a prominent risk factor associated with increased levels of distress and reduced mental well-being [6, 36]. Our findings underscore that sporting officials frequently contend with abusive behaviour from spectators, players, and coaches, which is linked to higher levels of symptoms of anxiety and depression [6, 36]. In addition, younger officials were found to be particularly vulnerable to the negative mental health impacts of abuse, reporting increased risk of quitting following such experiences [6, 36]. These findings underscore the benefit of individualised mental health support during the early stages of an official’s career to mitigate the negative impact of abuse and promote retention. As pointed out by previous research, if sporting organisations are not committing appropriate resources to reduce incidences of abuse, this may be a factor that contributes to the attrition of sporting officials [55].

Building on this, recent research by McKeen and Stevinson [56] involving current and former (thus, not meeting our inclusion criteria) sports officials expands the focus from solely reducing abuse to also encouraging more frequent positive interactions, such as compliments and verbal or written thanks. Results showed that regular positive exchanges were linked to lower likelihood of depression and anxiety symptoms. These results suggest that while minimising abuse is crucial, actively fostering ongoing, positive engagement with officials could be an effective strategy for enhancing mental health.

Considering the results around the association of age and level of experience, our review also highlights the need to foster access to early mental health support as well as to help reduce the likelihood of onset of distress. Extending on the findings of Carter et al.’s [7] scoping review, which highlighted the role of organisational culture in shaping mental health outcomes, our findings revealed a range of organisational factors (e.g. perceived lack of social support, stress from interpersonal conflicts, perceived lack of mental health training) to be associated with adverse mental health outcomes, such as higher symptoms of burnout, anxiety, along with increased intentions to quit officiating [6, 38, 41, 42, 50]. From the holistic ecological perspective [57], our findings highlight risk and protective factors at micro, exo- and macro-levels of the sport ecosystem [3]. Changes at the macrosystem level (i.e. [inter]national sporting organisation) may also help to facilitate microsystem changes (i.e. individual official), such as alignment between organisations' and officials' role expectations (e.g. role-culture) and changes in individual mental health outcomes. While our findings broadly align with those observed in athletes (e.g. [3, 19]), it is important to acknowledge that sporting officials operate within distinct environments and face unique stressors [14]. In light of these unique challenges, our review highlights the value of change or support at all levels of the official ecosystem that address the specific challenges of officiating to support mental health effectively.

While specific organisational policies relating to the support of officials' mental health and well-being may not always be publicly available, this does not necessarily mean that organisations do not have such policies in place. However, perceived organisational barriers reported by officials in previous studies [6, 35, 38] indicate that access to and utilisation of mental health services may present some challenges. The findings of this review suggest that organisations may seek to prioritise initiatives aimed at improving mental health literacy and fostering help-seeking behaviours, particularly in younger officials, who were found to experience higher rates of poor mental health outcomes and may lack the appropriate knowledge to access support. Thus, our findings underscore the benefit of sporting clubs and organisations extending existing mental health literacy in sport programs to officials. Higher levels of mental health literacy have the potential to help individuals to recognize mental health challenges early and access appropriate support, while an organisational culture that normalises help-seeking can aid in reducing stigma [30, 58]. Ensuring that existing well-being and mental health programs or services in sport environments are extended to sport officials is also important to respond to mental ill-health. Equipping officials with other protective skills, such as leadership skills, emotional regulation and adaptive coping skills may also confer additional benefit, complementing MHL training and mental health service access.

Addressing some of the specific organisational factors raised in this review could have important implications for shaping positive organisational culture change, such as viewing officials’ mental health as part of occupational health and safety requirements to increase awareness and promote sporting officials’ mental health widely across sport settings. Ensuring that organisational policies for safeguarding and promoting well-being include or consider officials, as well as athletes or other stakeholders in the sport system, is important. In addition, specific considerations for officials may also be needed. In particular, given the unique risk factor of abuse observed among sporting officials, continued refinement and enforcement of policies that reduce exposure to abuse are needed, as are strategies for minimising the impacts of abuse, including player/parent education and support options for officials. Further, engagement and control over officiating load were additional protective factors, whereas a lack of preparation and occupational stress were risk factors, pointing to a benefit of policies that enable agency and autonomy around role, scheduling and workload. In addition to addressing risk factors for sporting officials’ mental health, policies that further optimise the environment for officials can also play a role in promoting mental health and well-being. Systems for coaching or provision of feedback to officials vary across sporting organisations and levels, but structured coaching or feedback to support learning and development, especially of junior officials, may be another avenue to protect mental health. Coaching could be tailored to support feelings of preparation for officiating, mitigate concerns around performance and past decisions and enhance role competence.

Our findings indicate that female officials tend to report higher symptoms of depression and anxiety, and lower levels of psychological well-being relative to male officials [29, 34, 42, 47]. While caution is warranted given this was only directly reported in four studies, these findings are aligned with the widely reported sex disparity seen in symptoms of mental ill-health among athletes (e.g. [59, 60]). It is important to clarify that all the included studies reported gender and did not use gender terms when specifying participants within their samples. In addition, the review showed that research has predominantly been conducted on male officials, potentially neglecting important female-specific experiences in sports officiating, which should be a greater focus for the sport officiating literature in the future [61]. However, this disparity in sample demographics is largely reflective of a broader underrepresentation of female officials in sport, facilitated by structural barriers that make it more difficult for female officials to enter or remain in officiating roles [53]. Together, these findings indicate the need for greater focus on mental health research for females in sport (i.e., athletes, coaches and officials) to ensure these disparities are understood and targeted interventions can be implemented to address the specific needs of female officials [61].

### Limitations of the Extant Literature and Future Research Directions

A fundamental limitation of the extant literature included in this review is the heavy reliance on self-report measures to assess mental health outcomes, which previous research has shown to potentially overestimate poor mental health [62]. None of the included studies utilised diagnostic interviews, emphasizing the need for caution in interpreting the results. Another limitation is that the mental health disorder symptoms examined across studies were limited primarily to anxiety and depression (broadly defined). While study quality varied, a key limitation of studies reporting prevalence/incidence data was that no studies reported specific diagnostic criteria to differentiate between types of disorders (e.g. major depressive disorder vs persistent depressive disorder). Specific data on mental health symptoms (i.e. diagnostic criteria) would greatly enhance knowledge about the mental health challenges faced by sporting officials.

Another significant gap in the literature is the lack of longitudinal research, with only two longitudinal studies identified in this review [35, 38]. Thus, understanding of the long-term trajectory of mental health outcomes among officials and the impact of protective or risk factors over time remains limited. Further, there is a critical lack of studies investigating under-18 sporting officials, a group particularly vulnerable to mental health challenges [63, 64]. The present review also highlights a significant bias toward male samples. This bias not only reflects the underrepresentation of females in officiating roles but also restricts the understanding of female-specific experiences and mental health challenges in this population. Additional examination of mental health outcomes in underrepresented groups of officials, including risk and protective factors, and prevalence rates, will be beneficial for developing a comprehensive understanding of mental health in sporting officials across diverse demographics and life stages.

### Limitations of the Review

Methodologically, the review also faced several challenges. Minimal meta-analyses were conducted owing to heterogeneity in study designs and the unavailability of appropriate mental health symptom outcomes, limiting the ability to synthesize findings quantitatively. Further limitations of the review include significant heterogeneity in the prevalence rates of anxiety and depression, as well as the combining of symptom profiles (e.g. anxiety and depression) and limited detail on specific disorder types (e.g. eating disorders) in the included studies. In addition, potential publication bias was indicated in the analysis of depression prevalence rates. The presence of potential publication bias indicates that the published literature on mental health in officials may disproportionately reflect studies with elevated symptom profiles. Therefore, caution is needed when interpreting our results, as the observed prevalence may not fully represent the broader population of officials. Finally, most included studies consisted of male soccer officials aged 18 years and older, limiting our findings’ generalizability to other demographics, including female officials and those officiating non-soccer sports. An additional consideration is that only studies with active officials were included in the final review. While McKeen and Stevinson [56] included both active and retired officials in their population (and therefore did not meet the criteria for inclusion in this review), the majority (95%) were active officials, and no comparisons were made between active and current officials. Future research could provide a comparison between currently practicing officials and those no longer officiating.

One additional limitation is that officials are an extremely heterogeneous population, encompassing different sports, different sport values, different types of officials, in addition to varied sociological, ecological and historical contexts. Unfortunately, some generalisation is inevitable, and the role/environment of an Australian Rules football umpire will differ greatly to that of a boxing referee, for example. Furthermore, some characteristics of the role that could differ are the number of players, crowd size/behaviour, social norms of the sport and cultural factors. Therefore, readers should keep this in mind when interpreting the results, and future research should explore the nuances between officials from different sports, backgrounds and time periods.

## Conclusions

Sporting officials appear to experience similar rates of mental health symptoms- (anxiety and depression) compared with elite athlete populations. A range of personal and sport-environmental risk factors were identified, including potential impacts of younger age, lower level of officiating experience, socioeconomic challenges and experiences of verbal and/or physical abuse. Protective factors were largely the inverse of most risk factors but also indicate the potential influence of increased mental health literacy and help-seeking intentions for improved mental health outcomes. The results highlight that focus on change at all levels of the sport ecosystem may help to foster and promote positive mental health outcomes among sporting officials. The findings of this review available at that strategies tailored to officials could include age/level of experience-specific support interventions and creating organisational cultures that prioritise mental health outcomes.

## Supplementary Information

Below is the link to the electronic supplementary material.

Supplementary file1 (DOCX 85 KB)

Supplementary file2 (XLSX 52 KB)

## Declarations

### Data Availability

Data used in the present study are available in the supplementary material.
