# Supplementary material for: The Mental Health of Sporting Officials: A Systematic Review and Meta-analysis
Source: Sports Med. 2025 Sep 27;55(12):3059–91. doi: 10.1007/s40279-025-02315-1 (PMC12628492; doi:10.1007/s40279-025-02315-1)
Supplement: Supplementary file 1 — Supplementary file1 (DOCX 85 KB) [file 40279_2025_2315_MOESM1_ESM.docx]

**Supplemental Files**

**Appendix 1: PRISMA Checklist**

**Table 1**

| **Section and Topic** | **Item #** | **Checklist item** | **Location where item is reported** |
| --- | --- | --- | --- |
| **TITLE** | | |  |
| Title | 1 | Identify the report as a systematic review. | 1 |
| **ABSTRACT** | | |  |
| Abstract | 2 | See the PRISMA 2020 for Abstracts checklist. | 2 |
| **INTRODUCTION** | | |  |
| Rationale | 3 | Describe the rationale for the review in the context of existing knowledge. | 3-5 |
| Objectives | 4 | Provide an explicit statement of the objective(s) or question(s) the review addresses. | 5 |
| **METHODS** | | |  |
| Eligibility criteria | 5 | Specify the inclusion and exclusion criteria for the review and how studies were grouped for the syntheses. | 6-7 |
| Information sources | 6 | Specify all databases, registers, websites, organisations, reference lists and other sources searched or consulted to identify studies. Specify the date when each source was last searched or consulted. | 6 |
| Search strategy | 7 | Present the full search strategies for all databases, registers and websites, including any filters and limits used. | 6 |
| Selection process | 8 | Specify the methods used to decide whether a study met the inclusion criteria of the review, including how many reviewers screened each record and each report retrieved, whether they worked independently, and if applicable, details of automation tools used in the process. | 7 |
| Data collection process | 9 | Specify the methods used to collect data from reports, including how many reviewers collected data from each report, whether they worked independently, any processes for obtaining or confirming data from study investigators, and if applicable, details of automation tools used in the process. | 7-8 |
| Data items | 10a | List and define all outcomes for which data were sought. Specify whether all results that were compatible with each outcome domain in each study were sought (e.g. for all measures, time points, analyses), and if not, the methods used to decide which results to collect. | 6-8 |
|  | 10b | List and define all other variables for which data were sought (e.g. participant and intervention characteristics, funding sources). Describe any assumptions made about any missing or unclear information. | 6-8 |
| Study risk of bias assessment | 11 | Specify the methods used to assess risk of bias in the included studies, including details of the tool(s) used, how many reviewers assessed each study and whether they worked independently, and if applicable, details of automation tools used in the process. | 9 |
| Effect measures | 12 | Specify for each outcome the effect measure(s) (e.g. risk ratio, mean difference) used in the synthesis or presentation of results. | 9 |
| Synthesis methods | 13a | Describe the processes used to decide which studies were eligible for each synthesis (e.g. tabulating the study intervention characteristics and comparing against the planned groups for each synthesis (item #5)). | 7-9 |
|  | 13b | Describe any methods required to prepare the data for presentation or synthesis, such as handling of missing summary statistics, or data conversions. | 7-9 |
|  | 13c | Describe any methods used to tabulate or visually display results of individual studies and syntheses. | 7-9 |
|  | 13d | Describe any methods used to synthesize results and provide a rationale for the choice(s). If meta-analysis was performed, describe the model(s), method(s) to identify the presence and extent of statistical heterogeneity, and software package(s) used. | 9 |
|  | 13e | Describe any methods used to explore possible causes of heterogeneity among study results (e.g. subgroup analysis, meta-regression). | 9 |
|  | 13f | Describe any sensitivity analyses conducted to assess robustness of the synthesized results. | 9 |
| Reporting bias assessment | 14 | Describe any methods used to assess risk of bias due to missing results in a synthesis (arising from reporting biases). | NA |
| Certainty assessment | 15 | Describe any methods used to assess certainty (or confidence) in the body of evidence for an outcome. | NA |
| **RESULTS** | | |  |
| Study selection | 16a | Describe the results of the search and selection process, from the number of records identified in the search to the number of studies included in the review, ideally using a flow diagram. | Figure 1 |
|  | 16b | Cite studies that might appear to meet the inclusion criteria, but which were excluded, and explain why they were excluded. | Appendix 5 |
| Study characteristics | 17 | Cite each included study and present its characteristics. | Table 1 |
| Risk of bias in studies | 18 | Present assessments of risk of bias for each included study. | Tables 2 and 3 |
| Results of individual studies | 19 | For all outcomes, present, for each study: (a) summary statistics for each group (where appropriate) and (b) an effect estimate and its precision (e.g. confidence/credible interval), ideally using structured tables or plots. | Table 1 |
| Results of syntheses | 20a | For each synthesis, briefly summarise the characteristics and risk of bias among contributing studies. | 9-10 |
|  | 20b | Present results of all statistical syntheses conducted. If meta-analysis was done, present for each the summary estimate and its precision (e.g. confidence/credible interval) and measures of statistical heterogeneity. If comparing groups, describe the direction of the effect. | 23-24; Appendix 4 |
|  | 20c | Present results of all investigations of possible causes of heterogeneity among study results. | 23-24; Appendix 4 |
|  | 20d | Present results of all sensitivity analyses conducted to assess the robustness of the synthesized results. | 23-24 |
| Reporting biases | 21 | Present assessments of risk of bias due to missing results (arising from reporting biases) for each synthesis assessed. | NA |
| Certainty of evidence | 22 | Present assessments of certainty (or confidence) in the body of evidence for each outcome assessed. | 23-24; Appendix 4 |
| **DISCUSSION** | | |  |
| Discussion | 23a | Provide a general interpretation of the results in the context of other evidence. | 34-39 |
|  | 23b | Discuss any limitations of the evidence included in the review. | 39-40 |
|  | 23c | Discuss any limitations of the review processes used. | 40 |
|  | 23d | Discuss implications of the results for practice, policy, and future research. | 37-40 |
| **OTHER INFORMATION** | | |  |
| Registration and protocol | 24a | Provide registration information for the review, including register name and registration number, or state that the review was not registered. | 6 |
|  | 24b | Indicate where the review protocol can be accessed, or state that a protocol was not prepared. | 6 |
|  | 24c | Describe and explain any amendments to information provided at registration or in the protocol. | 6 |
| Support | 25 | Describe sources of financial or non-financial support for the review, and the role of the funders or sponsors in the review. | NA |
| Competing interests | 26 | Declare any competing interests of review authors. | NA |
| Availability of data, code and other materials | 27 | Report which of the following are publicly available and where they can be found: template data collection forms; data extracted from included studies; data used for all analyses; analytic code; any other materials used in the review. | Extracted data is reported in Table 1 and Table 4. All extracted data is available in supplementary material.  Template can be made available upon request. |

*From:*  Page MJ, McKenzie JE, Bossuyt PM, Boutron I, Hoffmann TC, Mulrow CD, et al. The PRISMA 2020 statement: an updated guideline for reporting systematic reviews. BMJ 2021;372:n71. doi: 10.1136/bmj.n71

**Appendix 2: Search String**

Full search string: (“mental” AND “illness” OR “mental” AND “disorder” OR “psych*” AND “problem” OR “depression” OR “anxiety” OR “stress”) OR (“mental” AND “health” OR “mental” AND “well-being” OR “mental” AND “wellbeing” OR “flourishing”) AND (“sport*”) AND (“referee” OR “umpire” OR “official”).

**Appendix 3: Inclusion and Exclusion criteria**

**Table 2**

| **Inclusion Criteria** | **Exclusion Criteria** |
| --- | --- |
| Presents original data | No original data |
| Quantitative designs | Reviews, positions statements, editorials, qualitative studies |
| English | Published in language other than English |
| Current sporting officials of any age and level of experience, defined as individuals engaged in officiating team or individual sports (i.e., referees, umpires, and judges). | Retired officials; studies which included activities such as dance or recreational fitness; non-officiating roles such as coaching or event management |
| Evaluation of mental health symptoms, disorders, and/or psychological wellbeing sustained over-time. | Acute/transient psychological conditions that are not directly linked to mental health (e.g., pre-competitive anxiety) |
| Valid assessment measures of mental health symptoms, mental health disorders or psychological well-being. | Unvalidated measures of mental health |

**Appendix 4: Meta-analysis Publication Bias**

***Publication bias - Anxiety***

***Figure 1: Trim-and-fill funnel plot to ascertain publication bias***

Non-shaded circles represent published effect sizes
Shaded circles represent imputed effect size

Non-shaded diamond represents the pooled effect size of published data
Shaded diamond represents the pooled effect size with n=0 imputed effect size

***Publication bias – Depression***

***Figure 2: Trim-and-fill funnel plot to ascertain publication bias***

Non-shaded circles represent published effect sizes
Shaded circles represent imputed effect size

Non-shaded diamond represents the pooled effect size of published data
Shaded diamond represents the pooled effect size with n=0 imputed effect size

**Appendix 5: List of studies excluded after full text review**

**Table 4**

| **Exclusion criteria** | **Reference** |
| --- | --- |
| No evaluation of mental health outcomes (n = 42) | Ackery, Tator (1); Aguilar, Castillo-Rodriguez (2); Aguirre-Loaiza, Holguín (3); Alsentali (4); Anshel, Sutarso (5); Antonie (6); Aragao e Pina, Passos (7); Arbinaga, Fernández-Ozcorta (8); Bernal, Nix (9); Bloß, Loffing (10); Louvet and Campo (11); Castillo-Rodríguez, López-Aguilar (12); Cuskelly and Hoye (13); Paula, Cunha (14); Demirtaş and Yıldız (15); Dhurup and Dubihlela (16);  Dodt, Fasold (17); Friesen, Devonport (18); Grylls, Turner (19); Hancock, Dawson (20); Kaissidis-Rodafinos and Anshel (21); Kaissidis-Rodafinos, Anshel (22); Kiss, Balogh (23); Kruger, Ekmekci (24); Kural and Aydin (25) Lee, Ko (26); Livingston and Forbes (27); Louvet, Gaudreau (28); Moen, Andersen (29); Nogueira, Fontes (30); Pizzera, Laborde (31); Pla-Cortés, Soriano-Gillué (32); Rainey (33); Samuel, Galily (34); Slack, Maynard (35); Stewart and Ellery (36); Stewart, Ellery (37); Voight (38); Warner, Tingle (39); Webb, Dicks (40); Wolfson and Neave (41); Gómez-López, Manzano-Sánchez (42) |
| Cannot retrieve full-text (n = 18) | Akdağcık (43); Ribeiro and Pires (44); Can, Çoban (45); Ceridono and Formica (46); Goldsmith and Williams (47); Gorczynski and Gibson (48); Heisterkamp (49); Hemmings and Graydon (50); Kaissidis-Rodafinos, Anshel (51); Li, Tsai (52); Louvet, Campo (53); Arjona, Coelho (54); Rahimi-Golkhandan, Ruddock-Hudson (55); Kim and Yip (56); Murakami, Sakata (57) ; Soriano, Ramis (58); Taylor, Daniel (59); Qian, Shuqin (60) |
| Studied transient psychological conditions not directly linked to mental health (n = 18) | Anshel, Kang (61); Anshel and Weinberg (62); Anshel and Weinberg (63); Castillo-Rodríguez, Muñoz-Arjona (64); Castillo-Rodríguez, Caparrós (65); Cullen, Clarke (66); Gencay (67); Hill, Matthews (68); Kaissidis and Anshel (69); Kovalchuk and Mospan (70); Munoz-Arjona and Castillo-Rodriguez (71); Philippe, Vallerand (72); Rainey and Hardy (73); Ritchie, Basevitch (74); Samuel, Englert (75); Tsorbatzoudis, Rodafinos (76); Downward, Webb (77); Houison, Lamont-Mills (78) |
| Full-text not available in English (n = 12) | Blasco (79); Burim and de Oliveira (80); GilluÉ, Laloux (81); Gullu and Yildiz (82); Jaenes, Bohórquez (83); Merino-Prados, Molero (84); Muñoz-Arjona, Fernandes (85); Pedrosa and García-Cueto (86); Pedrosa and García-Cueto (87); de Moura Simim, Ferreira (88); Sivri, Kiratli (89); Duran-Andrada, Khudhair (90) |
| Not original research (e.g., discussion paper, conference paper) (n = 11) | Bambaeichi, Movahedi (91); Carpenter (92); Davis and Stratton (93); Forbes and Livingston (94); Gorczynski and Webb (95), (96); Mellick (97); Schultz (98); Taylor and Daniel (99); Teques, Figueiredo (100); Huser (101) |
| Other research design [not quantitative] (n = 3) | Devís-Devís, Serrano-Durá (102); Radziszewski, Parent (103); Webb, Gorczynski (104) |
| Non-officiating population (n = 2) | Bu, Han (105); Jacobs, Tingle (106) |
| Not current officials | McKeen and Stevinson (107) |

**References**

1. Ackery AD, Tator CH, Snider C. Violence in Canadian amateur hockey: The experience of referees in Ontario. Clinical Journal of Sport Medicine. 2012;22(2):86-90.

2. Aguilar JL, Castillo-Rodriguez A, Chinchilla-Minguet JL, Onetti-Onetti W. Relationship between age, category and experience with the soccer referee’s self-efficacy. PeerJ. 2021;9:e11472.

3. Aguirre-Loaiza H, Holguín J, Arenas J, Núñez C, Barbosa-Granados S, García-Mas A. Psychological characteristics of sports performance: Analysis of professional and semiprofessional football referees. Journal of Physical Education and Sport. 2020;20(4):1861-8.

4. Alsentali A. Stressors and coping style among volleyball referees in the Northern Border region. International Journal of Advanced and Applied Sciences. 2022;9(12):68-76.

5. Anshel MH, Sutarso T, Ekmekci R, Saraswati IW. A model linking sources of stress to approach and avoidance coping styles of Turkish basketball referees. Journal of sports sciences. 2014;32(2):116-28.

6. Antonie A. Stressors in Romanian elite football refereeing–a comparison between 2009 and 2020. Journal of Physical Education and Sport. 2023;23(1):186-93.

7. Aragao e Pina J, Passos AM, Carvalho H, Travis Maynard M. To be or not to be an excellent football referee: Different experts’ viewpoints. Journal of Sports Sciences. 2019;37(6):692-700.

8. Arbinaga F, Fernández-Ozcorta EJ, Herrera-Macías PP, Vela-Calderón D. Burnout syndrome and resilience in soccer and basketball referees. Revista de psicologia del deporte. 2019;28(2):23-32.

9. Bernal J, Nix C, Boatwright D. Sport officials' longevity: motivation and passion for the sport. 2012.

10. Bloß N, Loffing F, Schorer J, Büsch D. Impact of psychological and physical load on the decision-making of top-class handball referees. International Journal of Performance Analysis in Sport. 2022;22(3):352-69.

11. Louvet B, Campo M. Do high emotional intelligent soccer referees better cope with competitive stressors? Movement & Sport Sciences-Science & Motricité. 2019(3):17-26.

12. Castillo-Rodríguez A, López-Aguilar J, Alonso-Arbiol I. Relationship between physical-physiological and psychological responses in amateur soccer referees. Revista de psicología del deporte. 2021;30(2):26.

13. Cuskelly G, Hoye R. Sports officials’ intention to continue. Sport Management Review. 2013;16(4):451-64.

14. Paula DAGd, Cunha RAd, Andreoli CV. Health problems of basketball referees: a prospective study. Revista Brasileira de Medicina do Esporte. 2021;27(2):195-200.

15. Demirtaş E, Yıldız K. FUTBOL HAKEMLERİNİN ÖZYETERLİLİK ALGILARI VE STRES FAKTÖRLERİNİN İŞ PERFORMANSLARINA ETKİSİNİN İNCELENMESİ. SPORMETRE Beden Eğitimi ve Spor Bilimleri Dergisi. 2021;19(4):157-69.

16. Dhurup M, Dubihlela D. Sport coaching officials and their stressors : work overload, role ambiguity, role conflict and the influence on job satisfaction of sport coaching officials in Gauteng province, South Africa : sport coaching. African Journal for Physical Health Education, Recreation and Dance. 2014;20(22):732-44.

17. Dodt M, Fasold F, Memmert D. Personality profile of team handball referees at expert level. German Journal of Exercise and Sport Research. 2022;52(1):58-67.

18. Friesen AP, Devonport TJ, Lane AM. Beyond the technical: The role of emotion regulation in lacrosse officiating. Journal of Sports Sciences. 2017;35(6):579-86.

19. Grylls E, Turner M, Erskine J. The Challenge of the Umpire’s Chair: Challenge and threat, self-efficacy, and psychological resilience in Australian tennis officials. International Journal of Sport Psychology. 2021.

20. Hancock D, Dawson D, Auger D. Why Ref? Understanding sport officials’ motivations to begin, continue, and quit. Movement & Sport Sciences - Science & Motricité. 2015:31-9.

21. Kaissidis-Rodafinos A, Anshel MH. Psychological predictors of coping responses among Greek basketball referees. The Journal of social psychology. 2000;140(3):329-44.

22. Kaissidis-Rodafinos A, Anshel MH, Porter A. Personal and situational factors that predict coping strategies for acute stress among basketball referees. Journal of Sports Sciences. 1997;15(4):427-36.

23. Kiss B, Balogh L, Münnich Á. A sport-psychological diagnostic examination of young EHF handball referees with a focus on mental skills. Journal of Physical Education & Sport. 2020;20(4).

24. Kruger A, Ekmekci R, Strydom G, Ellis S. Stressors among South African soccer officials: A profile analysis. South African Journal for Research in Sport, Physical Education and Recreation. 2012;34(2):53-62.

25. Kural S, Aydin F. Examining self-efficacy levels of football referees. Baltic Journal of Health and Physical Activity. 2021;13(7):12.

26. Lee P-C, Ko L-M, Chiu Y-C. The progression of Taiwanese women’s roles in officiating volleyball. The International Journal of the History of Sport. 2017;34(12):1275-93.

27. Livingston LA, Forbes SL. Factors contributing to the retention of Canadian amateur sport officials: Motivations, perceived organizational support, and resilience. International Journal of Sports Science & Coaching. 2016;11(3):342-55.

28. Louvet B, Gaudreau P, Menaut A, Genty J, Deneuve P. Revisiting the changing and stable properties of coping utilization using latent class growth analysis: A longitudinal investigation with soccer referees. Psychology of Sport and Exercise. 2009;10(1):124-35.

29. Moen C, Andersen TE, Clarsen B, Madsen-Kaarød G, Dalen-Lorentsen T. Prevalence and burden of health problems in top-level football Referees. Science and Medicine in Football. 2023;7(2):131-8.

30. Nogueira DFC, Fontes L, da Silva Gomes AR, da Silva RMCR. Emociones y percepción de rendimiento en árbitros: importancia del estrés y la evaluación cognitiva. Cuadernos de Psicología del Deporte. 2022;22(2):156-70.

31. Pizzera A, Laborde S, Lahey J, Wahl P. Influence of physical and psychological stress on decision-making performance of soccer referees. Journal of sports sciences. 2022;40(18):2037-46.

32. Pla-Cortés J, Soriano-Gillué G, Pérez-Guillorme AC, Soto-I-Mollfulleda P. Refereeing after COVID-19 lockdown: psychological effects on elite referees. 2023.

33. Rainey DW. Magnitude of stress experienced by baseball and softball umpires. Perceptual and Motor Skills. 1994;79(1):255-8.

34. Samuel RD, Galily Y, Tenenbaum G. Who are you, ref? Defining the soccer referee's career using a change-based perspective. International Journal of Sport and Exercise Psychology. 2017;15(2):118-30.

35. Slack LA, Maynard IW, Butt J, Olusoga P. Factors underpinning football officiating excellence: Perceptions of English Premier League referees. Journal of Applied Sport Psychology. 2013;25(3):298-315.

36. Stewart MJ, Ellery PJ. Sources and magnitude of perceived psychological stress in high school volleyball officials. Perceptual and Motor Skills. 1998;87(3_suppl):1275-82.

37. Stewart MJ, Ellery PJ, Ellery J, Maher L. Perceived psychological stress among high school basketball officials. Perceptual and Motor Skills. 2004;99(2):463-9.

38. Voight M. Sources of stress and coping strategies of US soccer officials. Stress and Health: Journal of the International Society for the Investigation of Stress. 2009;25(1):91-101.

39. Warner S, Tingle JK, Kellett P. Officiating attrition: The experiences of former referees via a sport development lens. Journal of Sport Management. 2013;27(4):316-28.

40. Webb T, Dicks M, Thelwell R, van Der Kamp J, Rix-Lievre G. An analysis of soccer referee experiences in France and the Netherlands: Abuse, conflict, and level of support. Sport Management Review. 2020;23(1):52-65.

41. Wolfson S, Neave N. Coping under pressure: Cognitive strategies for maintaining confidence among soccer referees. Journal of Sport Behavior. 2007;30(2):232-47.

42. Gómez-López M, Manzano-Sánchez D, Borrego CC, González-Hernández J. Perfectionism and fear of failure, according to sporting experience. A referee handball study. Frontiers in Psychology. 2025;Volume 16 - 2025.

43. Akdağcık İU. Voleybol hakemlerinin iş stres düzeylerinin farkli değişkenlere göre incelenmesi. Beden Eğitimi ve Spor Bilimleri Dergisi. 2023;17(1):1-9.

44. Ribeiro MVB, Pires DA. Percepção da síndrome de burnout em árbitros de futsal. Caderno de Educação Física e Esporte. 2019;17(2):65-9.

45. Can Y, Çoban Ü, Soyer F. NEGATİF DUYGUSALLIĞIN STRES ÜZERİNDEKİ ETKİSİ: FAAL FUTBOL HAKEMLERİ ÜZERİNDE BİR ARAŞTIRMA. Beden Eğitimi ve Spor Bilimleri Dergisi. 2011;5(2):165-74.

46. Ceridono D, Formica F. L’ansia negli arbitri di pallavolo. Movimento. 1987;3(2):140-2.

47. Goldsmith P, Williams JM. Perceived stressors for football and volleyball officials from three rating levels. Journal of Sport Behavior. 1992;15:106-18.

48. Gorczynski P, Gibson K. Words matter. 2018.

49. Heisterkamp G. Psychological aspects of the burden of the referee. Leistungssport. 1978;8(2):162-72.

50. Hemmings B, Graydon J. Sources of Stress and the Incidence of Burnout in National List and Junior League Status Football Referees in the 1992-93 Season. Journal of Sports Sciences. 1994;12:195.

51. Kaissidis-Rodafinos A, Anshel M, Sideridis G. Sources, intensity, and responses to stress in Greek and Australian basketball referees. 1998.

52. Li Y, Tsai W, Hsu Y, editors. Coping with an acute stress in sports-a study on athletes' perceived referee justice and emotional responses. INTERNATIONAL JOURNAL OF SPORT AND EXERCISE PSYCHOLOGY; 2021: ROUTLEDGE JOURNALS, TAYLOR & FRANCIS LTD 2-4 PARK SQUARE, MILTON PARK ….

53. Louvet B, Campo M, André A. Psychological determinants of coping strategies among soccer referees. Movement & Sport Sciences, 87, 63–77. 2015.

54. Arjona CM, Coelho BRF, Silva TDDS, Onetti WO, Rodríguez AC. Efecto de la experiencia del árbitro de fútbol en la competición deportiva. Cultura, ciencia y deporte. 2022;17(51):41-52.

55. Rahimi-Golkhandan S, Ruddock-Hudson M, Ruddock S. When the ball is bounced: Factors influencing the job of an AFL Umpire. Journal of Science and Medicine in Sport. 2019;22:S50.

56. Kim S, Yip WC. Sport referees' career commitment as a mediator between quality of work life (QWL) and turnover intent. International Journal of Applied Sports Sciences. 2018;30(2).

57. Murakami K, Sakata S, Matsuura M. A qualitative of examination coping strategies in international tennis umpires: Pilot study. Science and Racket Sports. 2019;6:91-7.

58. Soriano G, Ramis Y, Torregrosa M, Cruz J. Apoyo contra el estrés:¿ Cómo podemos ayudar a los árbitros. Kronos. 2017;16(1):1-10.

59. Taylor A, Daniel J, Leith L, Agnew J. An investigation of the psycho-social factors preceding dropout of Ontario soccer officials. Report to the Ontario Ministry of Tourism and Recreation Toronto, Canada. 1988.

60. Qian H, Shuqin C, Siyu M, Xiaoyu G, Yu Z, Bin Z, et al. Research on the occupational pressure of female referees in China Professional Football League. Journal of Chengdu Sport University. 2021;47(4):24-32.

61. Anshel MH, Kang M, Jubenville C. Sources of acute sport stress scale for sports officials: Rasch calibration. Psychology of Sport and Exercise. 2013;14(3):362-70.

62. Anshel MH, Weinberg RS. Re-examining coping among basketball referees following stressful events: Implications for coping interventions. Journal of Sport Behavior. 1999;22(2):141.

63. Anshel MH, Weinberg RS. Sources of acute stress in American and Australian basketball referees. Journal of Applied Sport Psychology. 1995;7(1):11-22.

64. Castillo-Rodríguez A, Muñoz-Arjona C, Onetti-Onetti W. National vs. Non-National Soccer Referee: Physiological, Physical, and Psychological Characteristics. Research Quarterly for Exercise and Sport. 2022;93(4):804-12.

65. Castillo-Rodríguez A, Caparrós JLR, Figueiredo A, González-Fernández FT, Onetti-Onetti W. Cause-Effect: The Relationship between Role and Experience with Psychological and Physical Responses in the Competition Context in Soccer Referees. Journal of Human Kinetics. 2023;89:289.

66. Cullen T, Clarke ND, Vaquera A. Sleep and mood of elite basketball referees during international competitions. Sport Sciences for Health. 2023;19(1):321-7.

67. Gencay S. Magnitude of psychological stress reported by soccer referees. Social Behavior and Personality: an international journal. 2009;37(7):865-8.

68. Hill DM, Matthews N, Senior R. The psychological characteristics of performance under pressure in professional rugby union referees. The Sport Psychologist. 2016;30(4):376-87.

69. Kaissidis A, Anshel M. Sources and intensity of acute stress in adolescent and adult Australian basketball referees: A preliminary study. Australian Journal of Science and Medicine in Sport. 1993;25:97-.

70. Kovalchuk V, Mospan M. Psychological component of the basketball referee's activity. Journal of Physical Education & Sport. 2020;20.

71. Munoz-Arjona C, Castillo-Rodriguez A. Attitude vs. Aptitude. Int J Sport Psychol. 2020;51:69-80.

72. Philippe FL, Vallerand RJ, Andrianarisoa J, Brunel P. Passion in referees: Examining their affective and cognitive experiences in sport situations. Journal of Sport and Exercise Psychology. 2009;31(1):77-96.

73. Rainey DW, Hardy L. Ratings of stress by rugby referees. Perceptual and Motor Skills. 1997;84(3):728-30.

74. Ritchie J, Basevitch I, Rodenberg R, Tenenbaum G. Situation criticality and basketball officials’ stress levels. Journal of Sports Sciences. 2017;35(21):2080-7.

75. Samuel RD, Englert C, Zhang Q, Basevitch I. Hi ref, are you in control? Self-control, ego-depletion, and performance in soccer referees. Psychology of Sport and Exercise. 2018;38:167-75.

76. Tsorbatzoudis H, Rodafinos A, Partemian S, Grouios G. Sources of, and responses to, stress among Greek team handball referees: Qualitative data. Journal of Human Movement Studies. 2005;49(4):297-314.

77. Downward P, Webb T, Dawson P. Referee Abuse, Intention to Quit, and Well-Being. Research Quarterly for Exercise and Sport. 2023.

78. Houison RJ, Lamont-Mills A, Kotiw M, Terry PC. They’re Only Human! Tension and Stress Predict Performance of Softball Umpires in National Championships. Sports [Internet]. 2025; 13(2).

79. Blasco T. Competencia personal, autoeficacia y estrés en árbitros de ciclismo. Revista de psicología del deporte. 1999;8(2):0195-205.

80. Burim ML, de Oliveira AR. Analysis of stress level of the futsal referee from the region of Londrina, Parana/ANALISE DO NIVEL DE ESTRESSE DOS ARBITROS DE FUTSAL DA REGIAO DE LONDRINA, PARANA. Revista Brasileira de Futsal e Futebol. 2018;10(38):252-62.

81. GilluÉ GS, Laloux YR, ÁLvarez MT. Sources of stress inside and outside the match in football referees. Apunts Educación Física y Deportes. 2018;34(132):22-31.

82. Gullu S, Yildiz SM. Stres Kaynaklarının Futbol Hakemlerinin Performansına Etkisinin İncelenmesi / Investigation of the Effect of the Stress Resources on Football Referees’ Performance. Ankara Üniversitesi Beden Eğitimi ve Spor Yüksekokulu SPORMETRE Beden Eğitimi ve Spor Bilimleri Dergisi. 2019;17:146-55.

83. Jaenes JC, Bohórquez MR, Caracuel JC, López A. Estado emocional y situaciones de estrés en árbitros de baloncesto. Cuadernos de psicología del deporte. 2012;12(2):17-24.

84. Merino-Prados H, Molero D, Gavín-Chocano Ó. Emotional intelligence, resilience, and life satisfaction in sport refereeing. 2023.

85. Muñoz-Arjona C, Fernandes BR, Dos Santos TD, Castillo-Rodríguez A. ¿ Influyen las respuestas psicológicas y la experiencia en el rendimiento físico del árbitro de fútbol? Aloma: revista de psicologia, ciències de l'educació i de l'esport Blanquerna. 2021;39(1):65-72.

86. Pedrosa I, García-Cueto E. Síndrome de Burnout en árbitros de élite: la liga de fútbol profesional española (LFP) a estudio. Revista Iberoamericana de Diagnóstico y Evaluación-e Avaliação Psicológica. 2016;2(42):59-68.

87. Pedrosa I, García-Cueto E. Aspectos psicológicos en árbitros de élite:¿ afecta el salario a su bienestar emocional? Revista de Psicología del Deporte. 2015;24(2):241-8.

88. de Moura Simim MA, Ferreira RM, Souza MVC, Marques AC, da Silva BVC. Psychological and social factors are subjective stress source in football referees/FATORES PSICOLOGICOS E SOCIAIS SAO FONTES DE ESTRESSE SUBJETIVO EM ARBITROS DE FUTEBOL. Revista Brasileira de Futsal e Futebol. 2018;10(39 SE):475-81.

89. Sivri S, Kiratli E, Karaca AA. Investigation of the relationship between psychological resilience and trait anxiety levels of tennis umpires. 2023.

90. Duran-Andrada J, Khudhair M, Ibarzábal F. Psychopathological symptoms in women football referees: A pilot study. Revista de Psicología Aplicada al Deporte y el Ejercicio Físico. 2025;10:e5,1-10.

91. Bambaeichi E, Movahedi AR, Abedini M. The relationship between cardiovascular risk factors and trait anxiety of Iranian referees and assistant referees in Premier League Soccer. British Journal of Sports Medicine. 2010;44(Suppl 1):i22-i.

92. Carpenter K. Extending the duty of care to achieve justice for abused match officials. The International Sports Law Journal. 2022;22(2):116-31.

93. Davis JA, Stratton MB. Effects of Gender and Rating Level on Trait Anxiety and Socialization Among Collegiate Volleyball Officials: 1430: Board# 169: May 28 9: 30 AM-11: 00 AM. Medicine & Science in Sports & Exercise. 2008;40(5):S211.

94. Forbes SL, Livingston LA. Changing the call: Rethinking attrition and retention in the ice hockey officiating ranks. Sport in Society. 2013;16(3):295-309.

95. Gorczynski P, Webb T. Developing a mental health research agenda for football referees. Soccer & Society. 2021;22(6):655-62.

96. Webb T, Gorczynski P. Factors influencing the mental health of sports match officials: The potential impact of abuse and a destabilised support system from a global context. Routledge Handbook of Athlete Welfare: Routledge; 2020. p. 289-99.

97. Mellick M. Soccer referee mental health: Developing a network of soccer referee mental health champions. The psychology of soccer: Routledge; 2020. p. 278-91.

98. Schultz TD. Comparisons of confidence and anxiety between high ability and low ability high school basketball officials. 1991.

99. Taylor A, Daniel J, editors. The construct validity of the Maslach Burnout Inventory in the sports role of officiating. Annual Conference for the North American Society for the Psychology of Sport and Physical Actvity Knoxville; 1988.

100. Teques P, Figueiredo P, Brito J, editors. Common mental disorders, coping strategies, and health-related behaviours among soccer referees. INTERNATIONAL JOURNAL OF SPORT AND EXERCISE PSYCHOLOGY; 2021: ROUTLEDGE JOURNALS, TAYLOR & FRANCIS LTD 2-4 PARK SQUARE, MILTON PARK ….

101. Huser R. Performance Enhancer: Mental Health Strategies are a Game Changer for Officials. Referee. 2023;4(48):34-6.

102. Devís-Devís J, Serrano-Durá J, Molina P. “The referee plays to be insulted!”: An exploratory qualitative study on the Spanish football referees’ experiences of aggression, violence, and coping. Frontiers in psychology. 2021;12:656437.

103. Radziszewski S, Parent S, St-Pierre E, Fortier J. “Never having the right to make a mistake, I think that’s the hardest part of being an official”: Exploring young sport officials’ experiences of abuse and their related coping mechanisms. Journal of Applied Sport Psychology. 2024;36(2):187-209.

104. Webb T, Gorczynski P, Oftadeh-Moghadam S, Grubb L. Experience and construction of mental health among English female football match officials. The Sport Psychologist. 2021;35(1):1-10.

105. Bu D, Han Z, Zhang C, Liu J, Huang Z, Liang W, et al. The effect of a mental health literacy intervention on Chinese team officials and staff in elite sports: a two-arm non-randomised controlled trial. International Journal of Sport and Exercise Psychology. 2024;22(9):2151-68.

106. Jacobs BL, Tingle JK, Oja BD, Smith MA. Exploring referee abuse through the lens of the collegiate rugby coach. Sport Management Review. 2020;23(1):39-51.

107. McKeen M, Stevinson C. The Frequency and Determinants of Positive and Negative Officiating Interactions and the Relationships with Mental Ill-Health Symptoms in Sports Officials. Sports Med. 2025;55(6):1527-42.
